# Development of a Highly Selective and Sensitive Fluorescent Probe for Imaging RNA Dynamics in Live Cells

**DOI:** 10.3390/molecules27206927

**Published:** 2022-10-15

**Authors:** Lan Fang, Wen Shao, Shu-Tang Zeng, Gui-Xue Tang, Jia-Tong Yan, Shuo-Bin Chen, Zhi-Shu Huang, Jia-Heng Tan, Xiu-Cai Chen

**Affiliations:** Guangdong Provincial Key Laboratory of New Drug Design and Evaluation, School of Pharmaceutical Sciences, Sun Yat-sen University, Guangzhou 510006, China

**Keywords:** RNA, RNA granules, small molecule, fluorescent probe, dynamics, cell imaging

## Abstract

RNA imaging is of great importance for understanding its complex spatiotemporal dynamics and cellular functions. Considerable effort has been devoted to the development of small-molecule fluorescent probes for RNA imaging. However, most of the reported studies have mainly focused on improving the photostability, permeability, long emission wavelength, and compatibility with live-cell imaging of RNA probes. Less attention has been paid to the selectivity and detection limit of this class of probes. Highly selective and sensitive RNA probes are still rarely available. In this study, a new set of styryl probes were designed and synthesized, with the aim of upgrading the detection limit and maintaining the selectivity of a lead probe **QUID−1** for RNA. Among these newly synthesized compounds, **QUID−2** was the most promising candidate. The limit of detection (LOD) value of **QUID−2** for the RNA was up to 1.8 ng/mL in solution. This property was significantly improved in comparison with that of **QUID−1**. Further spectroscopy and cell imaging studies demonstrated the advantages of **QUID−2** over a commercially available RNA staining probe, SYTO RNASelect, for highly selective and sensitive RNA imaging. In addition, **QUID−2** exhibited excellent photostability and low cytotoxicity. Using **QUID−2**, the global dynamics of RNA were revealed in live cells. More importantly, **QUID−2** was found to be potentially applicable for detecting RNA granules in live cells. Collectively, our work provides an ideal probe for RNA imaging. We anticipate that this powerful tool may create new opportunities to investigate the underlying roles of RNA and RNA granules in live cells.

## 1. Introduction

RNAs exhibit complex dynamics in live cells, including temporal and spatial processing and transportation [1]. The complex spatiotemporal dynamics of RNA molecules affect diverse cellular function. RNAs dynamically interact with a large group of RNA-binding proteins that modulate RNA localization and function [2]. Such RNA–protein interactions govern RNA expression, processing, export from the nucleus, and assembly into translationally competent messages, as well as association into translationally inactive RNA granules, including stress granules (SGs) and processing bodies (P-bodies) [3,4]. Given the intricate connection between RNA dynamics and function, it is highly desired to develop tools for visualization of RNA molecules in live cells to study their functions.

Although specific RNA targets in live cells have been successfully visualized by using protein-based [5] and oligonucleotide-based probes [6], the global dynamics of RNA distribution and the spatiotemporal processing and transportation of RNA molecules remains to be analyzed in live cells and in vivo. To achieve this goal, much attention has been paid to RNA-selective small-molecule fluorescent probes [7]. As a powerful tool to visualize dynamic cellular processes in living organisms, small-molecule fluorescent probes have shown many advantages in the monitoring of endogenous RNA dynamics in a convenient, continuous, and real-time way [8,9,10]. Unfortunately, RNA-selective small-molecule fluorescent probes suitable for RNA analysis are rarely available. To date, only one small-molecule fluorescent probe with RNA selectivity (named SYTO RNASelect) is commercially available. However, the practical applicability of SYTO RNASelect as an RNA-imaging probe in cells has been severely limited [11,12,13,14,15]. In addition, the molecular structure of SYTO RNASelect has not been published yet either, which makes it difficult to further improve the practical applicability based on SYTO RNASelect.

In this context, considerable effort has been devoted to the development of new small-molecule fluorescent probes for RNA imaging [11,12,13,14,15,16,17,18,19,20,21,22,23,24,25,26]. Until now, several fluorescent probes for intracellular RNA imaging have been developed, including styryl probes [13,15,25], crescent-shaped probe [20], V-shaped probe [26], and near-infrared probes [18,19]. Nevertheless, most of these studies mainly focused on improving the photostability, permeability, long emission wavelength, and compatibility with live-cell imaging of RNA probes. Less attention has been paid to the selectivity and detection limit of this class of probes despite the fact that these two factors are crucial for achieving RNA imaging selectively and sensitively. Indeed, highly selective and sensitive RNA probes for live-cell imaging have been rarely reported to date.

Recently, we discovered a novel RNA-selective fluorescent probe called **QUID−1** by enzyme-digestion-based screening in HeLa cells [27]. **QUID−1** is a quinoline-based indole-hemicyanine probe with a distinct turn-on response for RNA binding. Notably, **QUID−1** exhibited significant selectivity for RNA versus DNA, which was significantly better than the commercial probe, SYTO RNASelect (Figure S1). However, its detection limit for RNA is not satisfactory. The limit of detection (LOD) value of **QUID−1** for baker’s yeast RNA in solution was 26.5 ng/mL. Such data was much higher than data for commercial SYTO RNASelect with detection limit of 2.8 ng/mL for baker’s yeast RNA (Figure S2). These results impelled us to synthesize new probes based on **QUID−1** scaffold aiming to improve its detection limit and maintain its selectivity for RNA.

To develop highly selective and sensitive RNA probes, we herein designed and synthesized a new set of styryl probes based on **QUID−1** scaffold. Among these compounds, **QUID−2** was the most promising candidate. The limit of detection (LOD) value of **QUID−2** for the RNA was significantly improved in comparison with that of **QUID−1**. Further spectroscopy and cell imaging studies demonstrated the advantages of **QUID−2** over a commercially available RNA staining probe, SYTO RNASelect, for RNA imaging. Owing to the high selectivity, high sensitivity, low cytotoxicity, and excellent photostability of **QUID−2**, we obtained long-term monitoring of global dynamics of RNA. Moreover, **QUID−2** was found to have the potential to detect RNA granules in live cells.

## 2. Results and Discussion

### 2.1. Molecular Design of Probes **QUID−1**–**QUID−6**

**Figure 1:**
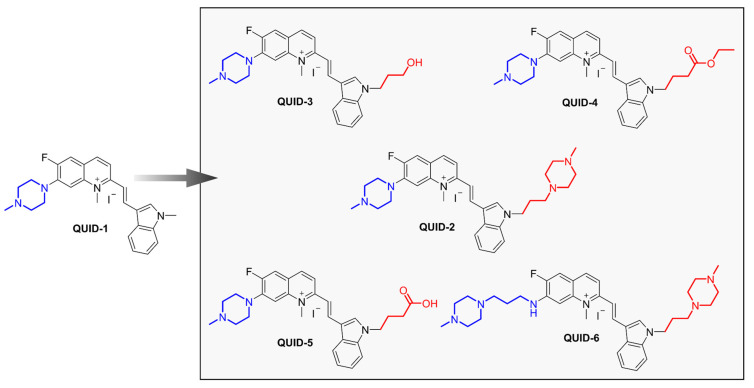
The structures of **QUID−1** and newly designed fluorescent probes **QUID−2** to **QUID−6**.

**QUID−1** was identified as a highly selective fluorescent probe for RNA in our previous studies [27]. However, its detection limit for RNA is not satisfactory. To develop highly selective and sensitive RNA probes, we attempted to synthesize a new set of analogues based on **QUID−1** scaffold to improve its detection limit and maintain its selectivity for RNA. The sensitivity of nucleic acid probes is caused by a high affinity for nucleic acids, in combination with a high quantum yield upon binding [28]. Thus, we envisaged the possibility of upgrading the detection limit of **QUID−1** by improving its binding affinity and fluorescence quantum yield with RNA. Previous studies indicated that the binding affinities between ligands and their target RNA often correlate with the number of cationic side chains present in the ligands [25,29,30]. Considering the structure of **QUID−1**, which includes a 1-methylpiperazine side group (p*K*a was calculated to be 8.99), this terminal cationic group makes an important contribution to the electrostatic interaction of **QUID−1** with RNA. However, this group alone is likely insufficient to achieve high-potency binding to RNA. On the basis of these results, we envisaged the improvement of probes’ binding affinity for RNA via introducing two cationic groups on the **QUID−1** scaffold. Therefore, another 1-methylpiperazine side group was introduced to the indole unit of **QUID−1**, yielding the resulting probe **QUID−2**. Three compounds named **QUID−3**, **QUID−4**, and **QUID−5** were also designed by replacing the 1-methylpiperazine group on the indole unit of **QUID−2** with OH, COOEt, and COOH, respectively, to determine the role of the cationic groups (1-methylpiperazine) in binding RNA (Figure 1). Because the carbon chain length of cationic group may sometimes influence the binding of probes to nucleic acid, **QUID−6** was designed by replacing the 1-methylpiperazine group on the quinolinium unit with 1-(3-aminopropyl)-4-methylpiperazine to explore the effect of the distance between 1-methylpiperazine group and the quinolinium unit on probe performance (Figure 1).

### 2.2. Synthesis of Fluorescent Probes **QUID−2**–**QUID−6**

**Scheme 1:**
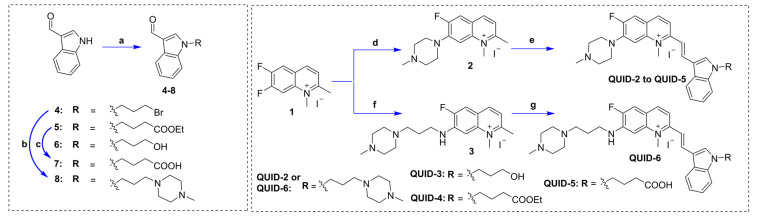
Synthesis of target compounds (**QUID−2** to **QUID−6**). Reagents and conditions: (a) alkyl bromides, acetonitrile, K_2_CO_3_, 80 °C, reflux; (b) 1-Methylpiperazine, acetonitrile, K_2_CO_3_, reflux; (c) i, 10% NaOH solution, reflux, ii, HCl; (d) 1-methylpiperazine, n-BuOH, 118 °C, 18 h; (e) intermediates 5–8, n-BuOH, 118 °C, 18 h; (f) 4-methyl-1-piperazinepropanamin, n-BuOH, 118 °C, 18 h; (g) intermediate 8, n-BuOH, 118 °C, 18 h.

The synthetic route for the probes (**QUID−2** to **QUID−6**) is shown in Scheme 1. Intermediate **1** was synthesized according to our previous report [27]. Regioselective nucleophilic substitution of the fluorine atom by 1-methylpiperazine or 1-(3-aminopropyl)-4-methylpiperazine gave the substitution intermediates **2** and **3**. The synthesis of intermediates **4**, **5**, and **6** began with commercially available indole-3-carboxaldehyde, which was dissolved in acetonitrile and reflux with 1,3-dibromopropane, ethyl 4-bromobutyrate, or 3-bromo-1-propanol in the presence of K_2_CO_3_. Intermediate **5** was hydrolyzed in 10% NaOH solution to give intermediate **7**. Intermediate **8** was synthesized from the nucleophilic substitution reaction of **4** with 1-methylpiperazine. The target compounds (**QUID−1** to **QUID−5**) were then prepared through the condensation of intermediate **2** and corresponding substituted indole-3-carboxaldehyde (intermediate **4** to intermediate **7**). **QUID−6** were prepared by condensing intermediate **3** and intermediate **8**. Their structures were confirmed by ^1^H and ^13^C NMR spectrometry, and HRMS spectrometry.

### 2.3. Screening **QUID** Fluorescent Probes

Upon synthesis, the spectroscopic properties of newly synthesized compounds were initially studied in vitro. These molecules structurally possess similar conjugation scaffold and thus they exhibited a slight difference in their absorption and emission maxima. The absorption maxima were found around 475 nm, and the emission maxima of the probes were found around 555 nm in TE buffer (Table 1 and Figure S3). Notably, these probes featured with the large Stokes shifts of ~80 nm, which was beneficial for RNA imaging in cells. To identify the most promising fluorescent probe for RNA imaging, newly synthesized compounds were screened for fluorescence performance with a focus on their detection limits and selectivity for RNA. Two representative nucleic acids, including baker’s yeast RNA and salmon testes DNA, were employed in the assays, as these nucleic acids from biological species are often utilized in studies for RNA-targeting fluorescent probes [13,15,31]. As shown in Table 1, these candidate probes alone in buffer displayed extremely weak emission, even in the presence of DNA samples. By contrast, most of the candidates displayed strong fluorescence when adding the RNA samples. As expected, candidates bearing two cationic side chains (**QUID−2** and **QUID−6**) exhibited a significantly better detection limit for RNA (Table 1 and Figure S4). However, the detection limits of **QUID−3**, **QUID−4**, and **QUID−5** are not satisfactory. Considering their structural features, **QUID−3**, **QUID−4**, and **QUID−5** contained terminal hydroxyl side chain, terminal ester side chain, and terminal carboxylic side chains, respectively. Neither of them was positively chargeable, thus demonstrating that the cationic amine side chain was indispensable. Among these candidates, **QUID−2** was the best probe in view of its outstanding detection limit for RNA. In addition, **QUID−2** displayed weaker fluorescence emission in the presence of DNA samples (fluorescence quantum yield *Φ*_F_ = 8.64%) relative to **QUID−6** (fluorescence quantum yield *Φ*_F_ = 19.33%), thus showing its better selectivity towards RNA. Among the candidates, **QUID−2** was chosen as the most promising RNA fluorescent probe for further detailed investigation.

### 2.4. Specificity Study of **QUID−2** towards RNA In Vitro

**Figure 2:**
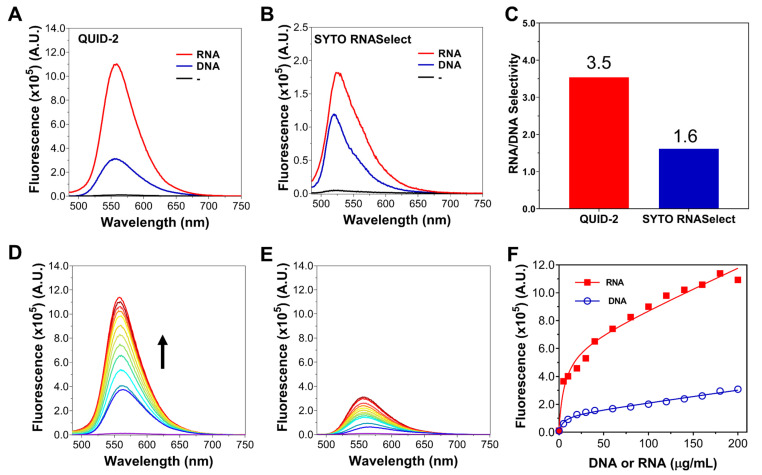
(**A**) Fluorescence emission spectrum of 1 μM **QUID−2** with or without 200 μg/mL baker’s yeast RNA or salmon testes DNA in TE buffer. (**B**) Fluorescence emission spectrum of 1 μM SYTO RNASelect with or without 200 μg/mL baker’s yeast RNA or salmon testes DNA in TE buffer. (**C**) The comparison of RNA/DNA selectivity of **QUID−2** and SYTO RNASelect. (**D**) The fluorescence titration of 1 μM **QUID−2** with the stepwise addition of baker’s yeast RNA (arrow: 0–200 μg/mL) in TE buffer. (**E**) The fluorescence titration of 1 μM **QUID−2** with the stepwise addition of salmon testes DNA (0–200 μg/mL) in TE buffer. (**F**) The fluorescence intensity enhancement of 1 μM **QUID−2** at 555 nm against the sample concentrations, *λ*_ex_ = 470 nm in TE buffer.

Given the promising results obtained above, we further evaluated the fluorescence response of **QUID−2** for RNA in detail. First, we investigated the fluorescence response of **QUID−2** (1.0 μM) to baker’s yeast RNA or salmon testes DNA (200 μg/mL) using fluorescence spectroscopy. As shown in Figure 2A, **QUID−2** displayed negligible emission in the absence of nucleic acid. This can be explained by the nonradiative energy loss through free rotation around the methine bridge between the indole and N-methylated quinoline moieties, as typically observed for various monomethine cyanine probes [32]. The addition of baker’s yeast RNA could significantly enhance the fluorescence signal of **QUID−2** at approximately 555 nm. In addition, we observed a hyperchromicity and a red shift of the absorption band of **QUID−2** when bound to RNA (Figure S5), which suggests the intercalative binding mode of **QUID−2** for RNA. Because **QUID−2** has little tendency to aggregate in a buffer (Figure S6), the observed turn-on response would arise from the suppressed rotation upon intercalation into the base pairs in the RNA. This assumption was further supported by the correlation between fluorescence intensity and solvent viscosity (Figure S7) [33].

The fluorogenic properties (I^RNA^/I^free^, where I^RNA^ and I^free^ denote the fluorescence intensities in the presence and absence of RNA, respectively) of **QUID−2** increased 120-fold upon binding to RNA, whereas an increase of only 34-fold was observed upon binding to DNA (I^DNA^/I^free^, where I^DNA^ and I^free^ denote the fluorescence intensities in the presence and absence of DNA, respectively). Notably, the value of RNA/DNA specificity (I^RNA^/I^DNA^) of **QUID−2** was higher than the commercial probe SYTO RNASelect (Figure 2A–C). These results suggest that **QUID−2** exhibited high selectivity toward RNA versus DNA. To further verify the selectivity of **QUID−2**, the fluorescent properties of **QUID−2** interacting with RNA and DNA were investigated by fluorescence titration. As shown in Figure 2D–F, with the gradual addition of the baker’s yeast RNA, an emission peak at approximately 555 nm was significantly enhanced. Nevertheless, negligible fluorescence enhancement was observed when titrating **QUID−2** with salmon testes DNA. Collectively, these results demonstrated that **QUID−2** specifically bind to RNA and showed much better RNA specificity than SYTO RNASelect.

### 2.5. Specificity Study of **QUID−2** towards RNA against Other Nucleic Acids in Cells

**Figure 3:**
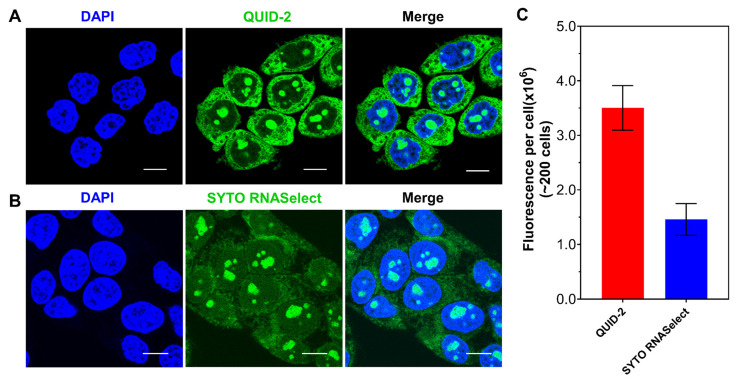
(**A**) Imaging of fixed HeLa cells stained with **QUID−2** (0.5 μM, *λ*_ex_ = 488 nm, *λ*_em_ = 500–600 nm, green). (**B**) Imaging of fixed HeLa cells stained with SYTO RNASelect (0.5 μM, *λ*_ex_ = 488 nm, *λ*_em_ = 500–600 nm, green). The nucleus was visualized by DAPI (*λ*_ex_ = 405 nm, *λ*_em_ = 430–490 nm, blue). (**C**) Quantification of the **QUID−2** and SYTO RNASelect fluorescence intensity for (**A**,**B**). For each sample, approximately 200 cells were measured, and the standard error was calculated from a set of three replicate experiments. Scale bars for cell image: 10 μm.

Encouraged by its desirable detection limit and specificity for RNA in vitro, we sought to evaluate the ability of **QUID−2** to specifically detect RNA in cells. Accordingly, confocal fluorescence microscopy was employed to assess the cellular imaging performance of **QUID−2**. Fixed HeLa cells were used in this test, and SYTO RNASelect was also tested as a control under identical measurement conditions ([probe] = 0.5 μM, 15 min incubation). As shown in Figure 3A, fixed HeLa cells stained with 0.5 μM **QUID−2** exhibited distinguishable bright nucleolar, cytoplasm staining, and faint nucleus. The staining patterns and distributions of **QUID−2** were very similar to that of SYTO RNASelect (Figure 3B) and other RNA fluorescent probes, suggesting that **QUID−2** selectively detect RNA in the cell. Notably, in sharp contrast to SYTO RNASelect, **QUID−2** displayed much brighter fluorescent emission in cells (Figure 3C). This is probably due to its superior selectivity and sensitivity for RNA over SYTO RNASelect.

**Figure 4:**
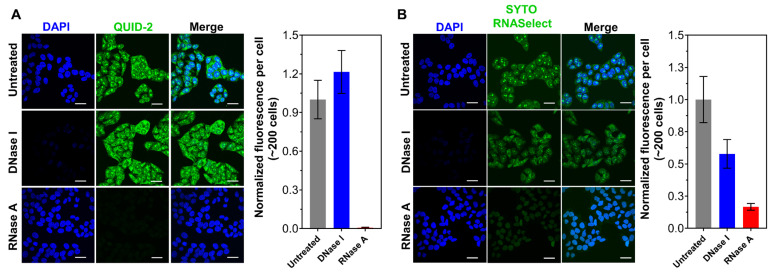
(**A**) Imaging of fixed HeLa cells stained with 0.5 μM **QUID−2** (*λ*_ex_ = 488 nm, *λ*_em_ = 500–600 nm, green) and treated by DNase I or RNase A. (**B**) Imaging of fixed HeLa cells stained with 0.5 μM SYTO RNASelect (*λ*_ex_ = 488 nm, *λ*_em_ = 500–600 nm, green) and treated by DNase I or RNase A. Quantification of the **QUID−2** and SYTO RNASelect fluorescence intensity is shown in the right panel. The nucleus was visualized by DAPI (*λ*_ex_ = 405 nm, *λ*_em_ = 430–490 nm, blue). For each sample, approximately 200 cells were measured, and the standard error was calculated from a set of three replicate experiments. Scale bars for cell image: 30 μm.

To further confirm the specificity of our probe towards RNA in cells, deoxyribonuclease (DNase) and ribonuclease (RNase) digestion experiments were carried out. In the DNase experiment, only the DNAs in the cell would be hydrolyzed. By contrast, in the RNase experiment, only RNAs would be hydrolyzed. Fixed-permeabilized HeLa cells were used in this test, and SYTO RNASelect was also selected as a control. As shown in Figure 4A, upon DNase I treatment, no fluorescence signal loss in either cytoplasm or nucleoli stained with **QUID−2** was observed. In contrast, upon RNase A treatment, the originally fluorescence signal from cytoplasm and nucleoli region that stained with **QUID−2** was dramatically disappeared. However, in the control group, the fluorescent signal of SYTO RNASelect after DNase I treatment was decreased because SYTO RNASelect also bound with DNA in cells (Figure 4B) [15]. These results evidently indicate that **QUID−2** is a more RNA-specific probe than SYTO RNASelect for imaging of RNA in cells.

### 2.6. Imaging of RNA Dynamics in Live Cells

Mounting evidence has demonstrated that RNAs exhibit complex dynamics in live cells and the complex spatiotemporal dynamics of RNA molecules affect diverse cellular function [34,35]. However, the global dynamics of RNA in live cells have rarely been studied, leading to an incomplete understanding of the complex spatiotemporal dynamics of RNA molecules. Therefore, we sought to explore the capacity of **QUID−2** in real-time monitoring the dynamics of RNA molecules in live cells.

**Figure 5:**
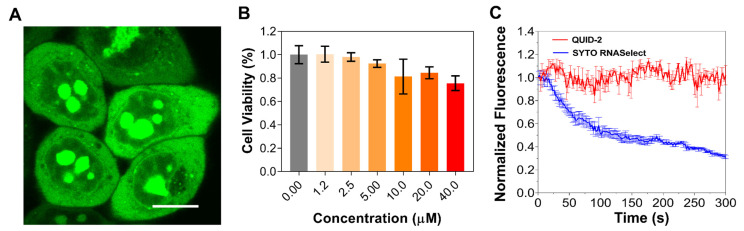
(**A**) Imaging of live HeLa cells stained with **QUID−2** (0.5 μM, *λ*_ex_ = 488 nm, *λ*_em_ = 500–600 nm, green). (**B**) Viability of HeLa cells exposed to different concentrations of **QUID−2** for 48 h determined by MTT assay. (**C**) Quantitative analysis of the changes in fluorescence intensities of **QUID−2** and SYTO RNASelect in live cells under continuous excitation at 488 nm. Scale bars for cell image: 10 μm.

Upon **QUID−2** treatment ([probe] = 0.5 μM, 30 min incubation), the live HeLa cells exhibited strong green fluorescent signal, with a distribution similar to that in fixed cells (Figure 5A), indicating that **QUID−2** selectively detect RNA in live cells. To ensure the practicability of **QUID−2** during long-term cell imaging, the cytotoxicity and photostability of the probe were further investigated. The cytotoxicity of **QUID−2** was evaluated in HeLa cells by standard MTT assay after 48 h of incubation. As shown in Figure 5B, **QUID−2** in the concentration range of 0 to 40 μM exerted negligible toxicity to HeLa cells, suggesting that **QUID−2** was safe at the working concentration of 0.5 μM for long term imaging in live cells. To study the photostability of **QUID−2** in live cells, continuous laser irradiation was applied over an extended period. SYTO RNASelect was also tested as a control under identical measurement conditions. After 300 s of continuous irradiation, the fluorescence signals of SYTO RNASelect decreased by 70%. By contrast, **QUID−2** did not show significant loss of the fluorescence signals (Figure 5C). These results indicate that **QUID−2** has satisfactory photostability and is suitable for long-term cell imaging.

**Figure 6:**
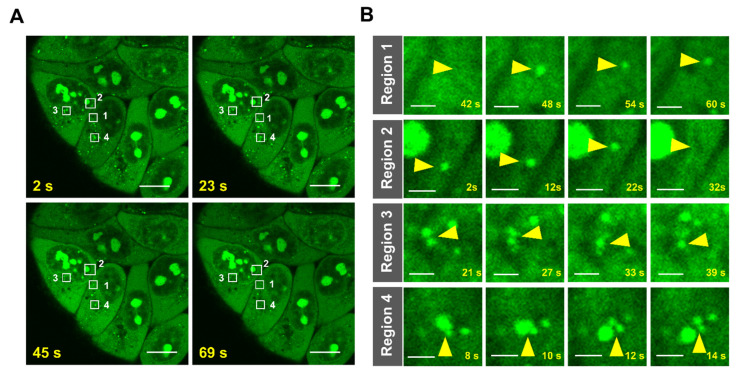
(**A**) Time-lapse imaging of RNA dynamics using **QUID−2** (0.5 μM, *λ*_ex_ = 488 nm, *λ*_em_ = 500–600 nm, green). (**B**) Images of the region denoted by the white boxes in (**A**). Scale bars in (**A**,**B**) are 10 and 2 μm, respectively.

**Figure 7:**
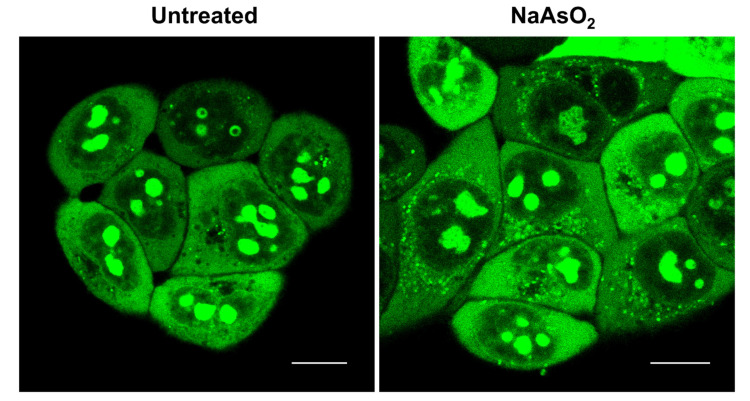
Imaging of live HeLa cells treated without or with sodium arsenite (500 μM) and stained with **QUID−2** (0.5 μM, *λ*_ex_ = 488 nm, *λ*_em_ = 500–600 nm, green). Scale bars for cell image: 10 μm.

Considering the favorable specificity, sensitivity, and performance of **QUID−2**, we then employed **QUID−2** to monitor the dynamics of RNA in live cells. Consistent with the dynamic nature of RNA [1,34,35], we observed complex spatiotemporal dynamics of **QUID−2** fluorescence signals in live HeLa cells (Figure 6A and Supplementary Materials Movie S1). In addition, we found some distinguishable bright **QUID−2** foci in the cytoplasm. These bright **QUID−2** foci were found to move in the cytoplasm, and several merging and splitting events were seen (Region 3, 4 in Figure 6A and Movie S1). Furthermore, we observed that during the measurement period some of the foci gradually disappeared, while new foci arose (Region 1, 2 in Figure 6A and Movie S1). This finding was similar to the results observed for RNA granules in live cells [36,37], suggesting that our probe might have the potential to detect RNA granules in live cells. To further test the practicability of using **QUID−2** to track the dynamics of RNA granules, arsenite (NaAsO_2_) was used to induce RNA granules (including SGs and P-bodies) formation [38]. As shown in Figure 7, upon exposure to arsenite (500 μM), **QUID−2** foci increased in both size and number. In addition, we compared the localization of **QUID−2** with BFP-G3BP1-labeled SGs (SGs are the most widely studied RNA granules and G3BP1 is a marker for SGs) in live HeLa cells treated with 500 μM sodium arsenite [36]. As expected, the fluorescent probe **QUID−2** accumulated in SGs and can be used to label SGs in live cells (Figure S8). Collectively, these results confirmed that **QUID−2** can detect RNA granules in live cells.

## 3. Conclusions

In summary, we have successfully developed a highly selective and sensitive fluorescent probe called **QUID−2** for RNA imaging through the structural modification of precursor probe **QUID−1**. The detection limit of **QUID−2** for the RNA was up to 1.8 ng/mL in solution. This value was significantly improved in comparison with its parent **QUID−1**, which had a detection limit of 26.5 ng/mL. We further demonstrated many advantages of **QUID−2** over a commercially available RNA staining probe, SYTO RNASelect, for selective and sensitive RNA sensing by a systematic comparison of fluorescent properties for RNA. Moreover, **QUID−2** possesses excellent photostability and low cytotoxicity in live cells, which is beneficial for long-term cell imaging. Thanks to many advantages of **QUID−2**, the global dynamics of RNA were revealed in live cells. More importantly, **QUID−2** was found to be potentially applicable for detecting RNA granules in live cells. Together, our work provides an ideal small-molecule probe for highly selective and sensitive RNA imaging. We anticipate that the further application of **QUID−2** in combination with biological and imaging methods to explore RNA and RNA granules dynamics in live cells will uncover more information about the biological roles of RNA molecules.

## 4. Materials and Methods

### 4.1. Materials

SYTO RNASelect (Thermo Fisher Scientific, S32703) was purchased from Thermo Fisher (Waltham, MA, USA). Salmon testes DNA (Sigma, D1626) and baker’s yeast RNA (Sigma, R6750) were purchased from Sigma (Saint Louis, MO, USA). RNase A and DNase I were purchased from Ambion (Austin, TX, USA). Hoechst 33,342 and DAPI were purchased from Sigma (Saint Louis, MO, USA). Sodium arsenite was purchased from Sigma (Saint Louis, MO, USA). TE buffer was purchased from Sangon Biotech (Shanghai, China). The HeLa cells used in this study were obtained from the cell bank of Sun Yat-Sen University Experimental Animal Center (Guangzhou, China).

### 4.2. Synthesis

^1^H and ^13^C NMR spectra were recorded on a Bruker Ascend^TM^ 400 or Ascend^TM^ 500 spectrometer (Karlsruhe, Germany). Mass spectra (MS) were recorded on a Shimadzu LCMS-2010A instrument (Tokyo, Japan) with an ESI or ACPI mass selective detector and high-resolution mass spectra (HRMS) were recorded on a Shimadzu LCMS-IT-TOF (Tokyo, Japan). Flash column chromatography was performed with silica gel (200–300 mesh) purchased from Qingdao Haiyang Chemical Co. Ltd. (Qingdao, China). All chemicals were purchased from commercial sources unless otherwise specified. All the solvents were of analytical reagent grade and were used without further purification. **QUID−1** was synthesized according to our previous report.

Synthesis of intermediate **4**: To a solution of indole-3-carboxaldehyde (4 g, 27 mmol) and anhydrous K_2_CO_3_ (3.8 g, 27 mmol) in acetonitrile (20.0 mL), 1,3-dibromopropane (28 g, 135 mmol) was added. The resulting mixture was heated to reflux for 12 h until the reaction was complete, and the solid was filtered away. After that, the remaining solution was concentrated under reduced pressure. The crude material was purified by column chromatography (DCM/PE 1:1) to give the product as colorless oil (**4**, 2.93 g, yield 40%). ^1^H NMR (400 MHz, Chloroform-*d*) δ 10.00 (s, 1H), 8.32 (d, *J* = 6.6 Hz, 1H), 7.77 (s, 1H), 7.41 (d, *J* = 7.0 Hz, 1H), 7.36–7.30 (m, 2H), 4.41 (t, *J* = 6.5 Hz, 2H), 3.35–3.31 (m, 2H), 2.44–2.37 (m, 2H). ESI-MS *m*/*z*: 267.0 [M + H]^+^.

Synthesis of intermediate **5**: To a solution of indole-3-carboxaldehyde (14.5 g, 100 mmol,) and anhydrous K_2_CO_3_ (13.8 g, 100 mmol) in acetonitrile (40.0 mL), ethyl 4-bromobutyrate (19.5 g, 100 mmol) was added. The resulting mixture was heated to reflux for 12 h until the reaction was complete, and the solid was filtered away. After that, the remaining solution was concentrated under reduced pressure. The crude material was purified by column chromatography (DCM/PE 1:1) to give the product as colorless oil (**5**, 16.6 g, yield 64%). ^1^H NMR (400 MHz, Chloroform-*d*) δ 9.94 (s, 1H), 8.34 (s, 1H), 8.14 (d, *J* = 7.6 Hz, 1H), 7.67 (d, *J* = 8.2 Hz, 1H), 7.37–7.27 (m, 2H), 4.34 (t, *J* = 7.1 Hz, 2H), 4.06–4.00 (m, 2H), 2.36 (t, *J* = 7.3 Hz, 2H), 2.13–2.06 (m, 2H), 1.16 (t, *J* = 7.1 Hz, 3H). ESI-MS *m*/*z*: 260.1 [M + H]^+^.

Synthesis of intermediate **6**: To a solution of indole-3-carboxaldehyde (1.45 g, 10 mmol,) and anhydrous K_2_CO_3_ (1.38 g, 10 mmol) in acetonitrile (5.0 mL), 3-bromo-1-propanol (1.39 g, 10 mmol) was added. The resulting mixture was heated to reflux for 12 h until the reaction was complete, and the solid was filtered away. After that, the remaining solution was concentrated under reduced pressure. The crude material was purified by column chromatography (DCM/PE 1.5:1) to give the product as colorless oil (**6**, 1.05 g, yield 52%). ^1^H NMR (400 MHz, DMSO-*d*_6_) δ 9.93 (s, 1H), 8.32 (s, 1H), 8.13 (d, *J* = 7.5 Hz, 1H), 7.65 (d, *J* = 8.1 Hz, 1H), 7.38–7.23 (m, 2H), 4.69 (t, *J* = 5.0 Hz, 1H), 4.37 (t, *J* = 7.0 Hz, 2H), 3.51–3.37 (m, 2H), 2.07–1.86 (m, 2H). ESI-MS 204.0 *m*/*z*: [M + H]^+^.

Synthesis of intermediate **7**: The compound **5** (2.6 g, 10 mmol) was dissolved in 20 mL 10% NaOH solution, and refluxed for 3 h (monitored by TLC). The mixture was allowed to cool to room temperature, then concentrated hydrochloric acid was added until the pH of the system was adjusted to about 3. The precipitate was filtered and dried under vacuum to afford white solid (1.39 g, 60%). ^1^H NMR (500 MHz, DMSO-*d*_6_) δ 12.21 (s, 1H), 9.90 (s, 1H), 8.31 (s, 1H), 8.10 (d, *J* = 7.8 Hz, 1H), 7.64 (d, *J* = 8.1 Hz, 1H), 7.34–7.23 (m, 2H), 4.30 (t, *J* = 7.1 Hz, 2H), 2.25 (t, *J* = 7.2 Hz, 2H), 2.07–1.98 (m, 2H). ESI-MS 232.0 *m*/*z*: [M + H]^+^.

Synthesis of intermediate **8**: To a solution of **4** (1.33 g, 5 mmol,) and anhydrous K_2_CO_3_ (0.69 g, 5 mmol) in acetonitrile (5.0 mL), 1-methylpiperazine (1 g, 10 mmol) was added. The resulting mixture was heated to reflux for 12 h until the reaction was complete, and the solid was filtered away. After that, the remaining solution was concentrated under reduced pressure. The crude material was purified by column chromatography (DCM/OH 80:1) to give the product as colorless oil (**8**, 0.63 g, yield 45%). ^1^H NMR (400 MHz, Chloroform-*d*) δ 10.01 (s, 1H), 8.30 (d, *J* = 7.5 Hz, 1H), 7.76 (s, 1H), 7.42 (d, *J* = 8.7 Hz, 1H), 7.35–7.30 (m, 2H), 4.28 (t, *J* = 6.7 Hz, 2H), 2.64–2.38 (m, 8H), 2.34–2.28 (m, 5H), 2.07–2.00 (m, 2H). ESI-MS 286.1 *m*/*z*: [M + H]^+^.

Synthesis of **QUID−2**: A mixture of **1** (64 mg, 0.19 mmol) and 1-methylpiperazine (40 mg, 0.37 mmol) in 1-butanol was heated to reflux for 12 h. Then intermediate **8** (79.8 mg, 0.28 mmol) was added for another 12 h under reflux. After cooling to room temperature, the mixture was filtered, and the precipitation was washed by ethanol and dried under vacuum to afford an orange red solid (**QUID−2**, 35 mg, yield 28%) ^1^H NMR (400 MHz, DMSO-*d*_6_) δ 8.62 (d, *J* = 8.8 Hz, 1H), 8.50–8.37 (m, 3H), 8.19 (d, *J* = 6.9 Hz, 1H), 8.04 (d, *J* = 13.1 Hz, 1H), 7.67 (d, *J* = 7.6 Hz, 1H), 7.59–7.45 (m, 2H), 7.38–7.27 (m, 2H), 4.42 (s, 3H), 4.37–4.31 (m, 2H), 3.54–3.32 (m, 4H), 2.81–2.55 (m, 9H), 2.45–2.24 (m, 11H), 2.04–1.96 (m, 2H). ^13^C NMR (126 MHz, DMSO-*d*_6_) δ 155.61, 153.56 (d, *J* = 253.3 Hz), 145.40 (d, *J* = 12.6 Hz), 140.27 (d, *J* = 3.3 Hz), 140.09, 137.60 (d, *J* = 4.8 Hz), 135.59, 125.93, 123.20, 121.96, 121.84, 120.33 (d, *J* = 4.1 Hz), 117.84, 114.51 (d, *J* = 23.9 Hz), 113.42, 112.20, 111.30, 106.03 (d, *J* = 1.8 Hz), 103.01, 54.07(2C), 53.78(2C), 53.27(2C), 49.34, 49.24(2C), 49.04, 45.09, 44.13, 40.00, 29.01. HRMS (ESI): calcd for (M-I)^+^ (C_33_H_42_FN_6_^+^) 541.3450, found 541.3399.

Synthesis of **QUID−3**: A mixture of **1** (64 mg, 0.19 mmol) and 1-methylpiperazine (40 mg, 0.37 mmol) in 1-butanol was heated to reflux for 12 h. Then intermediate **6** (57 mg, 0.28 mmol) was added for another 12 h under reflux. After cooling to room temperature, the mixture was filtered, and the precipitation was washed by ethanol and dried under vacuum to afford an orange red solid (**QUID−3**, 56 mg, yield 47.7%) ^1^H NMR (400 MHz, DMSO-*d*_6_) δ 8.64 (d, *J* = 8.9 Hz, 1H), 8.51–8.36 (m, 3H), 8.22 (d, *J* = 7.0 Hz, 1H), 8.06 (d, *J* = 13.1 Hz, 1H), 7.69 (d, *J* = 7.5 Hz, 1H), 7.62–7.46 (m, 2H), 7.45–7.27 (m, 2H), 4.72 (t, *J* = 5.0 Hz, 1H), 4.51–4.28 (m, 5H), 3.56–3.40 (m, 6H), 2.75–2.55 (m, 4H), 2.34 (s, 3H), 2.10–1.93 (m, 2H). ^13^C NMR (126 MHz, DMSO-*d*_6_) δ 155.54, 153.54 (d, *J* = 252.9 Hz), 145.48 (d, *J* = 9.9 Hz), 140.37 (d, *J* = 4.1 Hz), 139.97, 137.87, 137.53 (d, *J* = 5.2 Hz), 135.41, 125.96, 123.21, 121.98, 121.88, 120.26 (d, *J* = 4.3 Hz), 117.71, 114.46 (d, *J* = 23.3 Hz), 113.30, 112.09, 111.22, 105.90 (d, *J* = 3.1 Hz), 57.58, 54.15(2C), 49.34(2C), 45.49, 43.39, 40.11, 32.55. HRMS (ESI): calcd for (M-I)^+^ (C_28_H_32_FN_4_O^+^) 459.2555, found 459.2487.

Synthesis of **QUID−4**: A mixture of **1** (64 mg, 0.19 mmol) and 1-methylpiperazine (40 mg, 0.37 mmol) in 1-butanol was heated to reflux for 12 h. Then intermediate **5** (72.6 mg, 0.28 mmol) was added for another 12 h under reflux. After cooling to room temperature, the mixture was filtered, and the precipitation was washed by ethanol and dried under vacuum to afford an orange red solid (**QUID−4**, 62 mg, yield 48.3%) ^1^H NMR (400 MHz, DMSO-*d*_6_) δ 8.64 (d, *J* = 8.9 Hz, 1H), 8.49–8.33 (m, 3H), 8.21 (d, *J* = 7.1, 1H), 8.05 (d, *J* = 13.1 Hz, 1H), 7.70 (d, *J* = 8.8 Hz, 1H), 7.59–7.49 (m, 2H), 7.40–7.31 (m, 2H), 4.43 (s, 3H), 4.36 (t, *J* = 7.1 Hz, 2H), 4.05 (q, *J* = 7.1 Hz, 2H), 3.56–3.33 (m, 4H), 2.68–2.51 (m, 4H), 2.38 (t, *J* = 7.3 Hz, 2H), 2.31 (s, 3H), 2.15–2.08 (m, 2H), 1.17 (t, *J* = 7.1 Hz, 3H). ^13^C NMR (126 MHz, DMSO-*d*_6_) δ 172.16, 155.53, 153.57 (d, *J* = 252.8 Hz), 145.46 (d, *J* = 9.4 Hz), 140.46 (d, *J* = 3.4 Hz), 139.86, 137.88, 137.19 (d, *J* = 5.4 Hz), 135.16, 125.92, 123.27, 121.93, 121.87, 120.35 (d, *J* = 4.4 Hz), 117.75, 114.56 (d, *J* = 22.7 Hz), 113.46, 112.33, 111.16, 105.89 (d, *J* = 3.9 Hz), 60.00, 54.16(2C), 49.35(2C), 49.32, 45.47, 40.11, 30.56, 24.96, 14.04. HRMS (ESI): calcd for (M-I)^+^ (C_31_H_36_FN_4_O_2_^+^) 515.2817, found 515.2751.

Synthesis of **QUID−5**: A mixture of **1** (64 mg, 0.19 mmol) and 1-methylpiperazine (40 mg, 0.37 mmol) in 1-butanol was heated to reflux for 12 h. Then intermediate **7** (64.7 mg, 0.28 mmol) was added for another 12 h under reflux. After cooling to room temperature, the mixture was filtered, and the precipitation was washed by ethanol and dried under vacuum to afford an orange red solid (**QUID−5**, 45 mg, yield 36.6%) ^1^H NMR (400 MHz, DMSO-*d*_6_) δ 12.22 (s, 1H), 8.64 (d, *J* = 8.9 Hz, 1H), 8.47–8.37 (m, 3H), 8.22 (d, *J* = 7.0 Hz, 1H), 8.05 (d, *J* = 13.2 Hz, 1H), 7.71 (d, *J* = 7.4 Hz, 1H), 7.60–7.51 (m, 2H), 7.41–7.33 (m, 2H), 4.44 (s, 3H), 4.37 (t, *J* = 7.2 Hz, 2H), 3.50–3.45 (m, 4H), 2.62–2.57 (m, 4H), 2.35–2.28 (m, 5H), 2.15–2.03 (m, 2H). ^13^C NMR (126 MHz, DMSO-*d*_6_) δ 173.77, 155.52, 153.57 (d, *J* = 252.8 Hz), 145.53 (d, *J* = 10.0 Hz), 140.44 (d, *J* = 3.5 Hz), 139.86, 137.21, 137.53 (d, *J* = 5.1 Hz), 135.12, 125.94, 123.28, 121.98, 121.87, 120.32 (d, *J* = 4.9 Hz), 117.71, 114.46 (d, *J* = 23.5 Hz), 113.42, 112.30, 111.17, 105.83 (d, *J* = 3.7 Hz), 54.26(2C), 49.49(2C), 49.45, 45.63, 45.57, 30.62, 25.04. HRMS (ESI): calcd for (M-I)^+^ (C_29_H_32_FN_4_O_2_^+^) 487.2504, found 487.2451.

Synthesis of **QUID−6**: A mixture of **1** (64 mg, 0.19 mmol) and 1-(3-aminopropyl)-4-methylpiperazine (58.8 mg, 0.37 mmol) in 1-butanol was heated to reflux for 12 h. Then intermediate **8** (79.8 mg, 0.28 mmol) was added for another 12 h under reflux. After cooling to room temperature, the mixture was filtered, and the precipitation was washed by ethanol and dried under vacuum to afford an orange solid (**QUID−6**, 22 mg, yield 15%) ^1^H NMR (400 MHz, DMSO-*d*_6_) δ 8.36 (d, *J* = 8.6 Hz, 1H), 8.25–8.00 (m, 3H), 7.96 (d, *J* = 8.6 Hz, 1H), 7.77 (d, *J* = 16.2 Hz, 1H), 7.67 (d, J = 10.3 Hz, 1H), 7.54–7.28 (m, 2H), 7.28–7.04 (m, 2H), 6.87 (d, *J* = 6.7 Hz, 1H), 4.37–3.98 (m, 5H), 3.09–2.94 (m, 2H), 2.57–2.21 (m, 18H), 2.21–1.99 (m, 10H), 1.88–1.70 (m, 2H). ^13^C NMR (126 MHz, DMSO-*d*_6_) δ 155.00, 153.47 (d, *J* = 263.3 Hz), 143.65 (d, *J* = 14.4 Hz), 140.78 (d, *J* = 3.5 Hz), 139.73, 138.02 (d, *J* = 6.2 Hz), 137.27, 125.79, 123.03, 121.59, 120.28, 119.20 (d, *J* = 9.8 Hz), 117.90, 115.03 (d, *J* = 27.6 Hz), 113.10, 112.71, 112.39, 111.17 (d, *J* = 5.8 Hz), 95.07, 56.20, 54.62(4C), 54.34, 52.63(2C), 52.41(2C), 45.64, 45.57, 44.14, 41.89, 40.02, 26.69, 24.16. HRMS (ESI): calcd for (M-I)^+^ (C_36_H_49_FN_7_^+^) 598.4028, found 598.3974.

### 4.3. pKa Calculations

The p*K*a of 1-methylpiperazine side group was calculated using ChemBioDraw 14.0.

### 4.4. UV-Vis Absorption Studies

UV-Vis absorption studies were performed on a UV-2600 spectrophotometer (Shimadzu, Tokyo, Japan) using 1 cm path length quartz cuvette and the UV-Vis spectra in the range of 200–750 nm were recorded.

### 4.5. Fluorescence Studies

Fluorescence studies were performed on Fluoromax-4 Spectrofluorometer (HORIBA, Kyoto, Japan). A quartz cuvette with 2 mm × 10 mm path length was used for the spectra recorded at 5 nm excitation and emission slit widths unless otherwise specified. For the emission spectra of **QUID−1**–**QUID−6** or SYTO RNASelect (1 μM) in TE buffer (pH 7.8–8.2), the fluorescence spectra in the range of 485–750 nm were recorded when excited at 470 nm. For titration experiments, small aliquots of a stock solution of baker’s yeast RNA or salmon testes DNA were added into the solution containing probes (1 μM) in TE buffer (pH 7.8–8.2). After each addition of sample, the reaction was stirred and allowed to equilibrate for at least 2 min. The fluorescence spectra in the range of 485–750 nm was recorded when excited at 470 nm. The LOD values of probes for baker’s yeast RNA in solution were calculated on the basis of the equation LOD = *K* × *S*_b_/*m*. The *K* value is generally taken to be 3 according to the IUPAC recommendation. The *S*_b_ value represents the standard deviation for multiple measurements (*n* = 20) of blank solution. The *m* value is the slope of the calibration curve, which was derived from the linear range of a probe fluorescence titration curve with baker’s yeast RNA and standards for the sensitivity of this method. The absolute fluorescence quantum yield (*Φ*_F_) of **QUID−1**–**QUID−6** was measured by Quanta-Phi module for Fluoromax-4 Spectrofluorometer.

### 4.6. Fixed Cell Staining Experiments

The HeLa cells were grown in MEM media containing 10% fetal bovine serum and cultured at 37 °C in a CO_2_/air (5%/95%) incubator. Cells were seeded in a glass-bottom 96-well plate (Thermo Fisher Scientific) and grew overnight. Cells were fixed with 4% paraformaldehyde in DEPC-PBS at room temperature for 15 min. After being washed with PBS for 5 min (3×), cells were stained with 0.5 μM probe (**QUID−2** or SYTO RNASelect) and 0.5 μg mL^−1^ DAPI for 15 min at 37 °C. For deoxyribonuclease (DNase) and ribonuclease (RNase) digestion experiments, cells were permeabilized with 0.5% Triton X-100 in DEPC-PBS at 37 °C for 20 min, and then incubated with 200 units mL^−1^ enzymes (RNase A or DNase I) before staining. Samples were finally imaged on an FV3000 laser scanning confocal microscope (Olympus, Tokyo, Japan) with a 60× objective lens. The emission of probes (**QUID−2** and SYTO RNASelect) was collected under excitation at 488 mm. The images were analyzed with Imaris software (Bitplane, Zurich, Switzerland).

### 4.7. Cell Viability Assay

The HeLa cells were grown in MEM media containing 10% fetal bovine serum and cultured at 37 °C in a CO_2_/air (5%/95%) incubator. HeLa cells were seeded in 96-well plates (5.0 × 10^3^ cells per well) and exposed to various concentrations of **QUID−2**. After 48 h treatment, 20 μL of 2.5 mg/mL methylthiazolyl tetrazolium (MTT) solution was added to each well, and cells were further incubated for 4 h. The cells in each well were then treated with DMSO (100 μL per well), and the optical density (OD) was recorded at 490 nm. All experiments were performed in parallel and in triplicate, and the IC_50_ values were derived from the curves of the mean OD values of the triplicate tests plotted against the drug concentration.

### 4.8. Plasmid Transfection

cDNAs encoding BFP-G3BP1 were obtained by gene synthesis from Convenience Biology (Changzhou, China). To generate BFP-tagged G3BP1 protein, cDNAs were inserted into the pcDNA3.1 vector and the construct was confirmed by sequencing. Plasmid transfection of cells was performed using Lipofectamine 3000 (Invitrogen, Carlsbad, CA, USA) according to the manufacturer’s instructions. Then, 100 ng of constructed plasmid were used per well of a glass bottom 96-well plate (Thermo Fisher Scientific) and incubated at 37 °C with CO_2_ for 24 h.

### 4.9. Live Cell Staining Experiments

The HeLa cells were grown in MEM media containing 10% fetal bovine serum and cultured at 37 °C in a CO_2_/air (5%/95%) incubator. Cells were seeded in a glass-bottom 96-well plate (Thermo Fisher Scientific) and grew overnight. The cells were then stained with **QUID−2** (0.5 μM) in FluoroBrite DMEM (Gibco, A1896701) in a 5% CO_2_ atmosphere at 37 °C for 20 min, and then rinsed by FluoroBrite DMEM. For sodium arsenite treatment, cells were treated with (500 μM) sodium arsenite for 1 h before imaging. For co-staining with BFP-G3BP1, HeLa cells expressing BFP-G3BP1 were stained with 0.5 μM **QUID−2** for 20 min and then stressed with 500 μM sodium arsenite for 1 h. Digital images were recorded using a FV3000 laser scanning confocal microscope (Olympus, Tokyo, Japan) with a 60× objective lens and analyzed with Imaris software (Bitplane, Zurich, Switzerland). The emission of **QUID−2** was collected under excitation at 488 mm.

## Figures and Tables

**Table 1:** The optical data, limit of detection (LOD) value of probes (**QUID−1**–**QUID−6**) for baker’s yeast RNA, and fluorescence quantum yields (*Φ*_F_) of probes (**QUID−1**–**QUID−6**) in the presence of salmon testes DNA or baker’s yeast RNA.

Compound	*λ*_abs_ (nm)	*λ*_em_ (nm)	LOD (ng/mL)	*Φ*_F_^a^ (%)		
				Buffer	DNA	RNA
**QUID−1**	478	560	26.5	0.45	6.58	16.55
**QUID−2**	475	555	1.8	0.61	8.64	36.37
**QUID−3**	475	556	24.8	0.90	10.83	18.61
**QUID−4**	475	558	44.6	0.17	2.51	15.80
**QUID−5**	474	556	65.0	0.63	5.25	15.31
**QUID−6**	470	549	10.7	1.71	19.33	36.63

^a^ 1 μM of each compound and 100 μg/mL of DNA or RNA were used in the determination of *Φ*_F_.

## Data Availability

Data are contained within the article or Supplementary Materials.

## Supplementary Materials

The following supporting information can be downloaded at: https://www.mdpi.com/article/10.3390/molecules27206927/s1, Figure S1: Selectivity of **QUID−1** or SYTO RNASelect toward RNA versus DNA; Figure S2: Linear fit equations for calculating LOD values of **QUID−1** and SYTO RNASelect; Figure S3: Normalized UV and fluorescence emission spectrum of **QUID−1**–**QUID−6**; Figure S4: Linear fit equations for calculating LOD values of probes (**QUID−2**–**QUID−6**); Figure S5: The UV spectra of **QUID−2** with or without RNA; Figure S6: Concentration-Dependent UV-Vis absorbance of **QUID−2**; Figure S7: The spectra of **QUID−2** in glycerol-water mixed solution; Figure S8: Co-staining **QUID−2** with BFP-G3BP1; Figures S9–S28: ^1^H NMR spectrum, ^13^C NMR spectrum, and HRMS spectrum of newly synthesized compounds; Movie S1: Imaging of RNA dynamics in live HeLa cells s using **QUID-2**.

## Sample Availability

The samples of the compounds used and/or analyzed during the current study are available from the corresponding author upon reasonable request.
