# Supplementary material for: Development of a Highly Selective and Sensitive Fluorescent Probe for Imaging RNA Dynamics in Live Cells
Source: Molecules. 2022 Oct 15;27(20):6927. doi: 10.3390/molecules27206927 (PMC9607629; doi:10.3390/molecules27206927)
Supplement: Supplementary file 1 [file molecules-27-06927-s001.zip › FL_Molecules_SI.pdf]

Supplementary Materials

Development of a Highly Selective and Sensitive Fluorescent Probe for Imaging RNA Dynamics in Live Cells

Lan Fang†, Wen Shao†, Shu-Tang Zeng, Gui-Xue Tang, Jia-Tong Yan, Shuo-Bin Chen, Zhi-Shu Huang, Jia-Heng Tan and Xiu-Cai Chen*

Guangdong Provincial Key Laboratory of New Drug Design and Evaluation, School of Pharmaceutical Sciences, Sun Yat-sen University, Guangzhou, 510006, China

* E-mail address: chenxc35@mail.sysu.edu.cn (Xiu-Cai Chen).

†These authors contributed equally to this work.

Table of Contents:

Figure S1. Selectivity of **QUID−1** or SYTO RNASelect toward RNA versus DNA S3

Figure S2. Linear fit equations for calculating LOD values of **QUID−1** and SYTO RNASelect S4

Figure S3. Normalized UV and fluorescence emission spectrum of **QUID−1**−**QUID−6** S4

Figure S4. Linear fit equations for calculating LOD values of probes (**QUID−2**−**QUID−6**) S5

Figure S5. The UV spectra of **QUID−2** with or without RNA S5

Figure S6. Concentration-Dependent UV-Vis absorbance of **QUID−2** S6

Figure S7. The spectra of **QUID−2** in glycerol-water mixed solution S6

Figure S8. Co-staining **QUID−2** with BFP-G3BP1 S7

Figure S9. ^{1}H NMR spectrum of intermediate **4** S7

Figure S10. ^{1}H NMR spectrum of intermediate **5** S8

Figure S11. ^{1}H NMR spectrum of intermediate **6** S8

Figure S12. ^{1}H NMR spectrum of intermediate **7** S9

Figure S13. ^{1}H NMR spectrum of intermediate **8** S9

Figure S14. ^{1}H NMR spectrum of **QUID−2** S10

Figure S15. ^{13}C NMR spectrum of **QUID−2** S10

Figure S16. HRMS spectrum of **QUID−2** S11

Figure S17. ^{1}H NMR spectrum of **QUID−3** S12

Figure S18. ^{13}C NMR spectrum of **QUID−3** S12

Figure S19. HRMS spectrum of **QUID−3** S13

Figure S20. ^{1}H NMR spectrum of **QUID−4** S14
Figure S21. ^{13}C NMR spectrum of **QUID−4** S14
Figure S22. HRMS spectrum of **QUID−4** S15
Figure S23. ^{1}H NMR spectrum of **QUID−5** S16
Figure S24. ^{13}C NMR spectrum of **QUID−5** S16
Figure S25. HRMS spectrum of **QUID−5** S17
Figure S26. ^{1}H NMR spectrum of **QUID−6** S18
Figure S27. ^{13}C NMR spectrum of **QUID−6** S18
Figure S28. HRMS spectrum of **QUID−6** S19

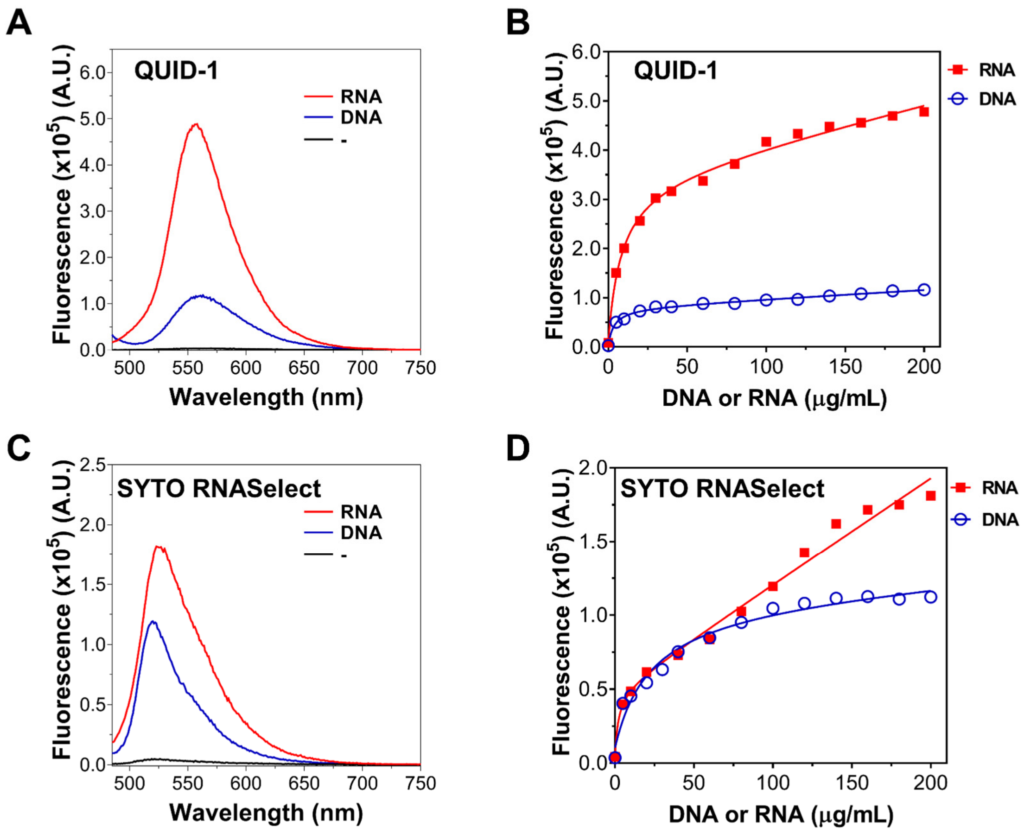

Figure S1. Selectivity of **QUID−1** or SYTO RNASelect toward RNA versus DNA. (A) Fluorescence emission spectrum of 1 μM **QUID−1** with or without 200 μg/mL baker's yeast RNA or salmon testes DNA in TE buffer. (B) The fluorescence intensity enhancement of 1 μM **QUID−1** at 560 nm against the sample concentrations, λ_{ex} = 470 nm. (C) Fluorescence emission spectrum of 1 μM SYTO RNASelect with or without 200 μg/mL baker's yeast RNA or salmon testes DNA in TE buffer. (D) The fluorescence intensity enhancement of 1 μM SYTO RNASelect at 525 nm against the sample concentrations, λ_{ex} = 470 nm.

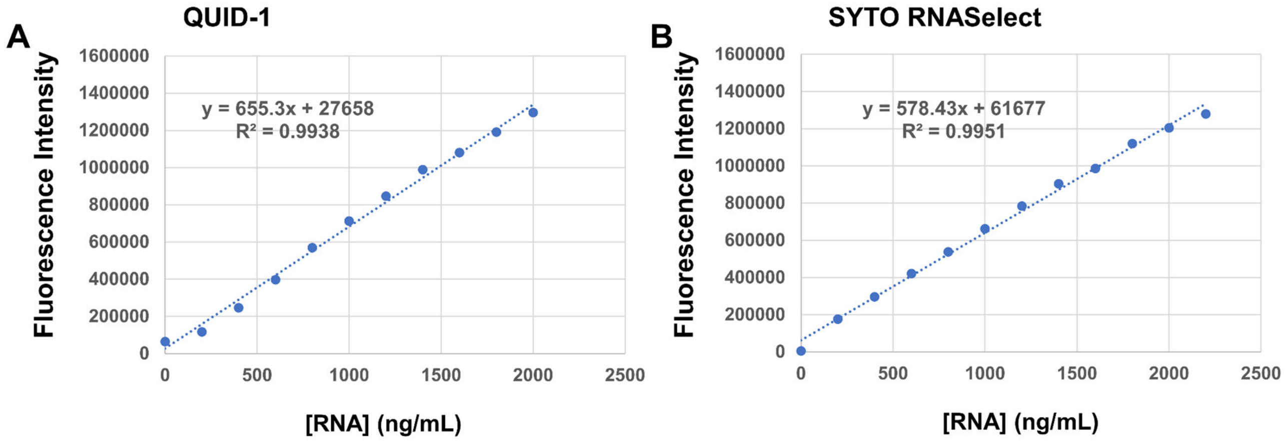

Figure S2. (A) Linear fit equations for calculating LOD values of **QUID−1** for baker's yeast RNA. (B) Linear fit equations for calculating LOD values of SYTO RNASelect for baker's yeast RNA.

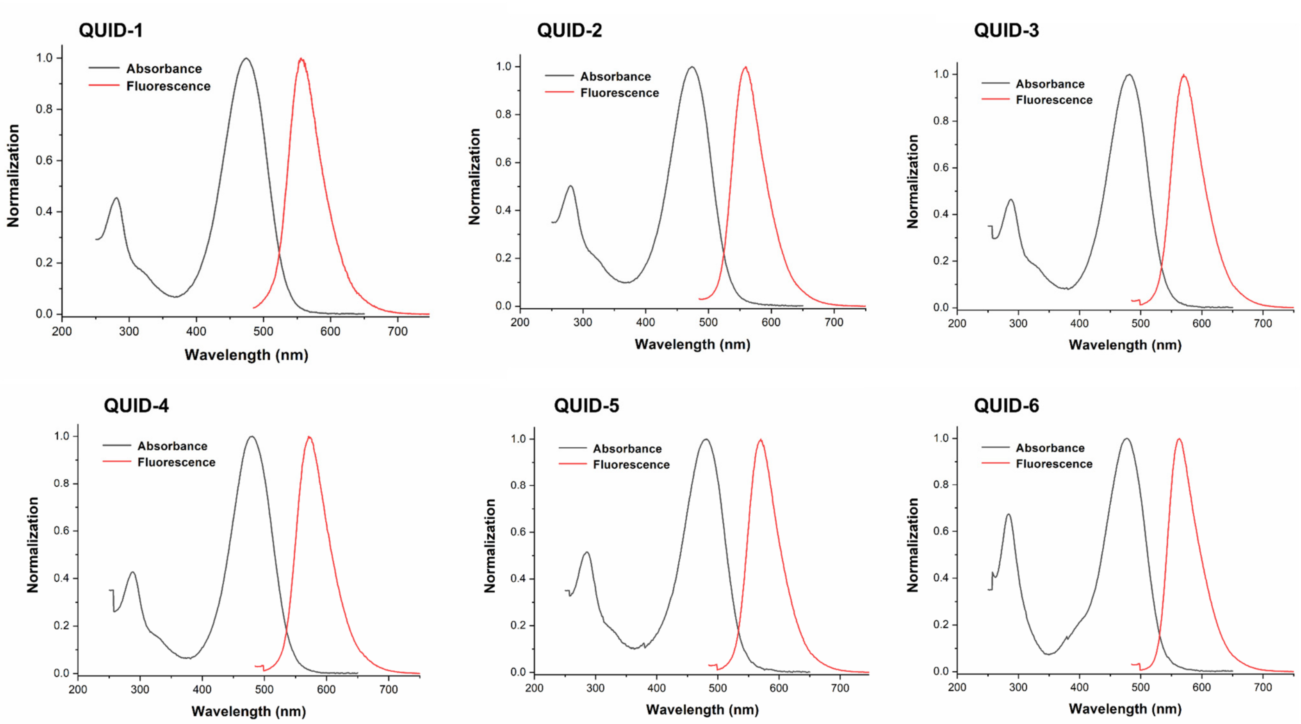

Figure S3. Normalized UV spectra of **QUID−1**−**QUID−6** (5 μM) in TE buffer and normalized fluorescence emission spectrum of **QUID−1**−**QUID−6** (1 μM) with 100 μg/mL baker's yeast RNA.

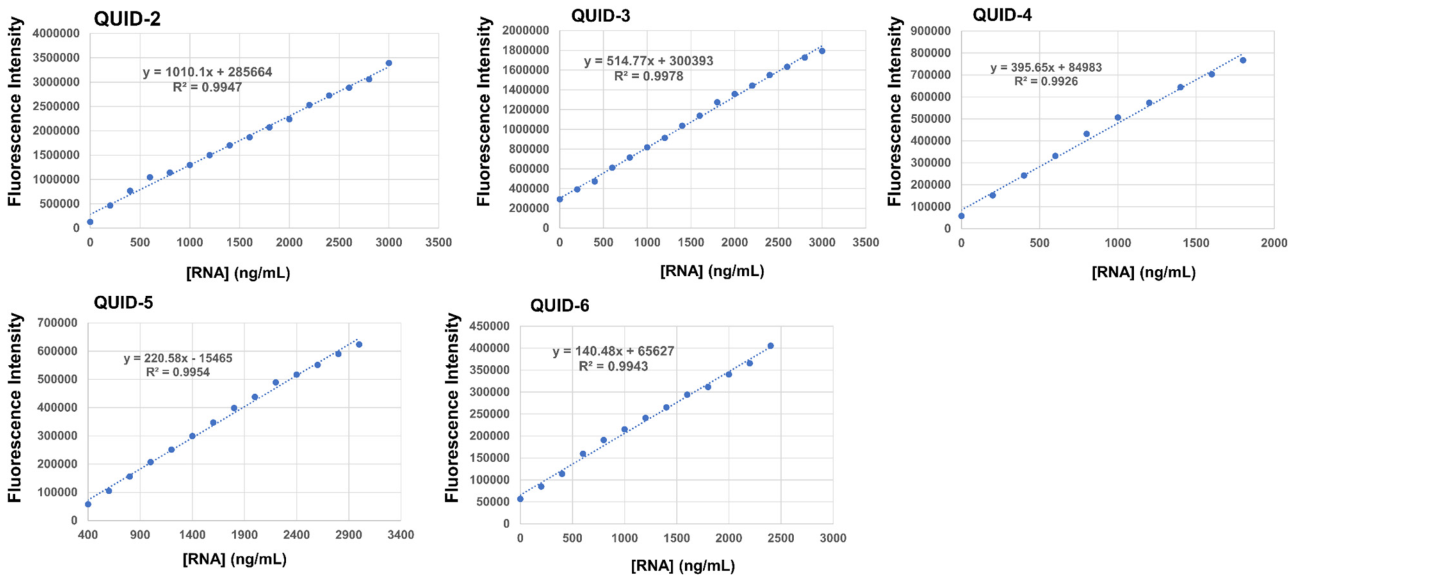

Figure S4. Linear fit equations for calculating LOD values of probes (**QUID−2**−**QUID−6**) for baker's yeast RNA.

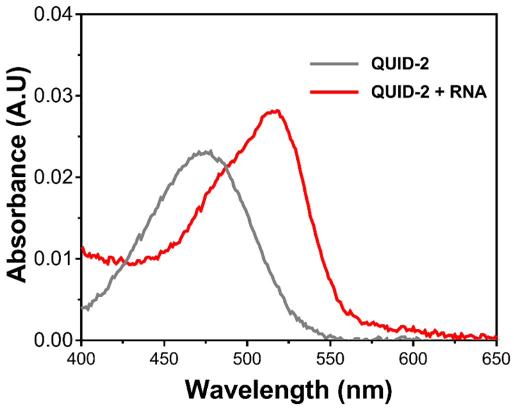

Figure S5. The UV spectra of **QUID−2** (1 μM) with or without 100 μg/mL baker's yeast RNA in TE buffer.

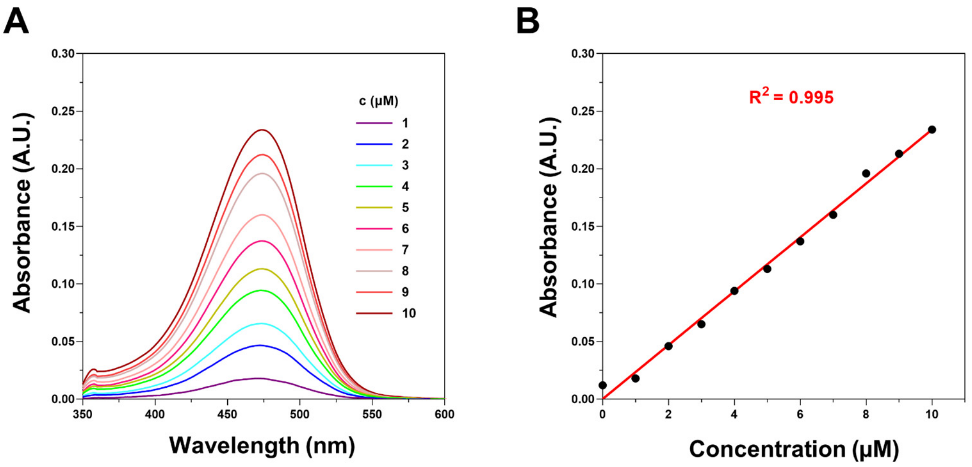

Figure S6. Concentration-Dependent UV-Vis absorbance of **QUID−2**. All of the spectra were collected in TE buffer. (A) The spectra of **QUID−2** at different concentrations. (B) The absorbance of **QUID−2** at 475 nm showed that the variations follow the Beer-Lambert law. **QUID−2** had little propensity to aggregate in buffer according to its concentration dependent absorbance spectra.

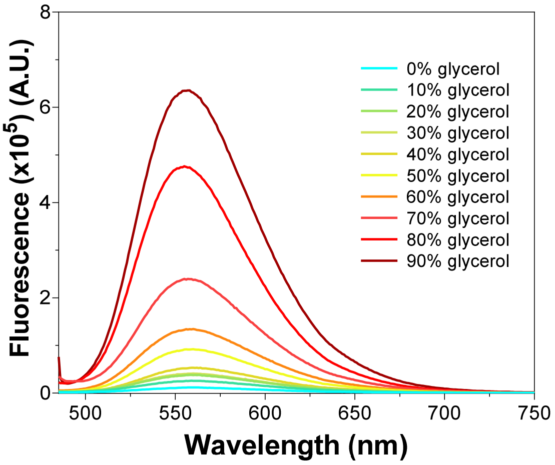

Figure S7. The spectra of **QUID−2** (1 μM) in glycerol-water mixed solution.

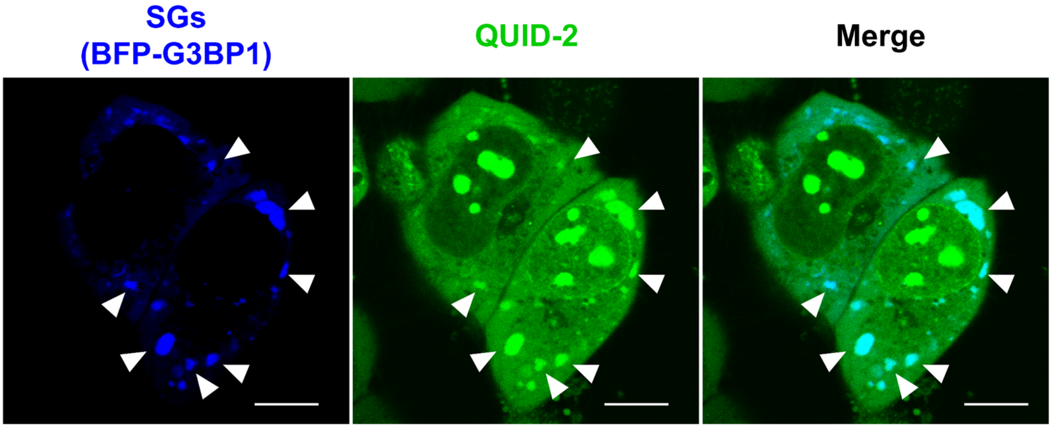

Figure S8. Co-staining **QUID−2** (0.5 μM, λ_{ex} = 488 nm, λ_{em} = 500-600 nm, green) with BFP-G3BP1 (λ_{ex} = 405 nm, λ_{em} = 430-480 nm, blue) in live cells treated with sodium arsenite (500 μM). Scale bars for cell image: 10 μm. White arrowheads indicate SGs.

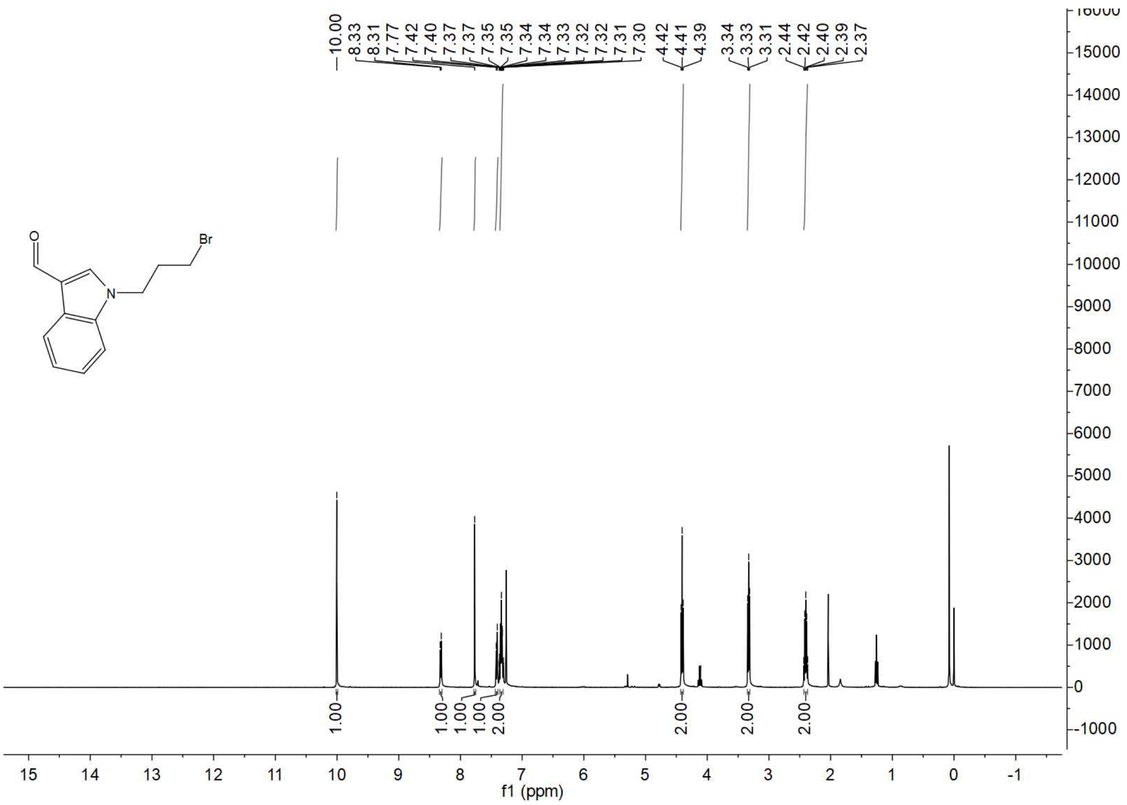

Figure S9. ^{1}H NMR spectrum of intermediate **4**.

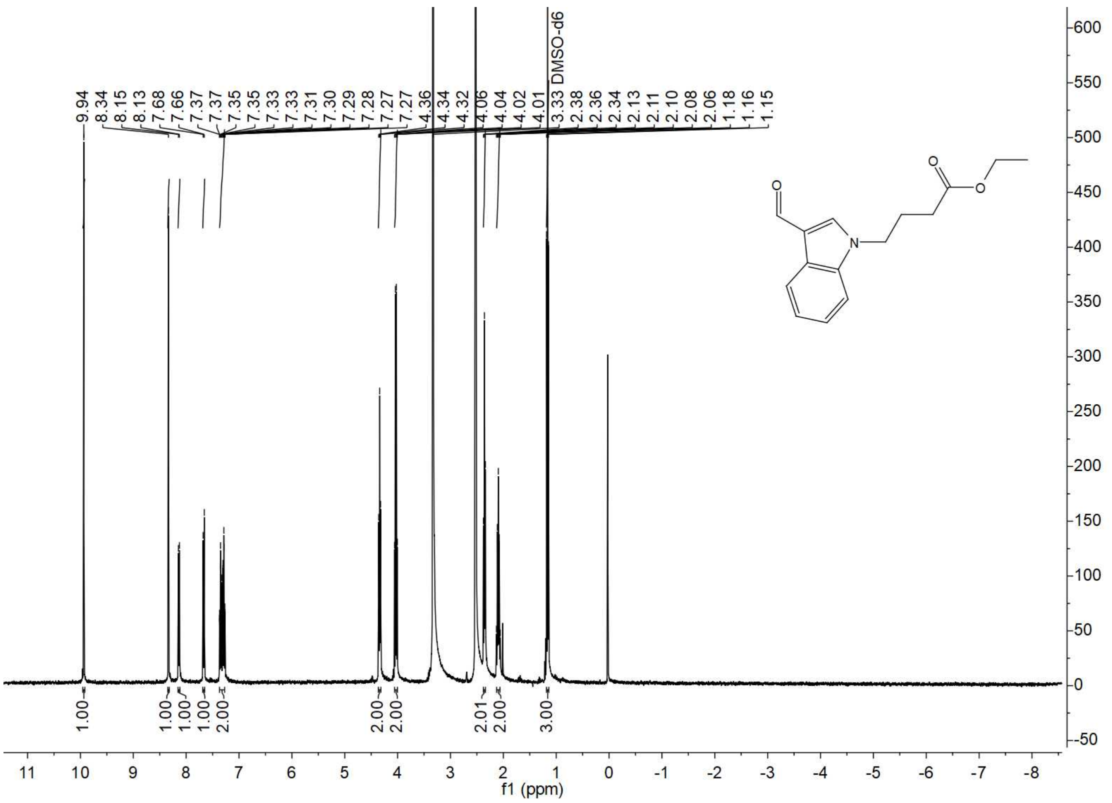

Figure S10. ^{1}H NMR spectrum of intermediate **5.**

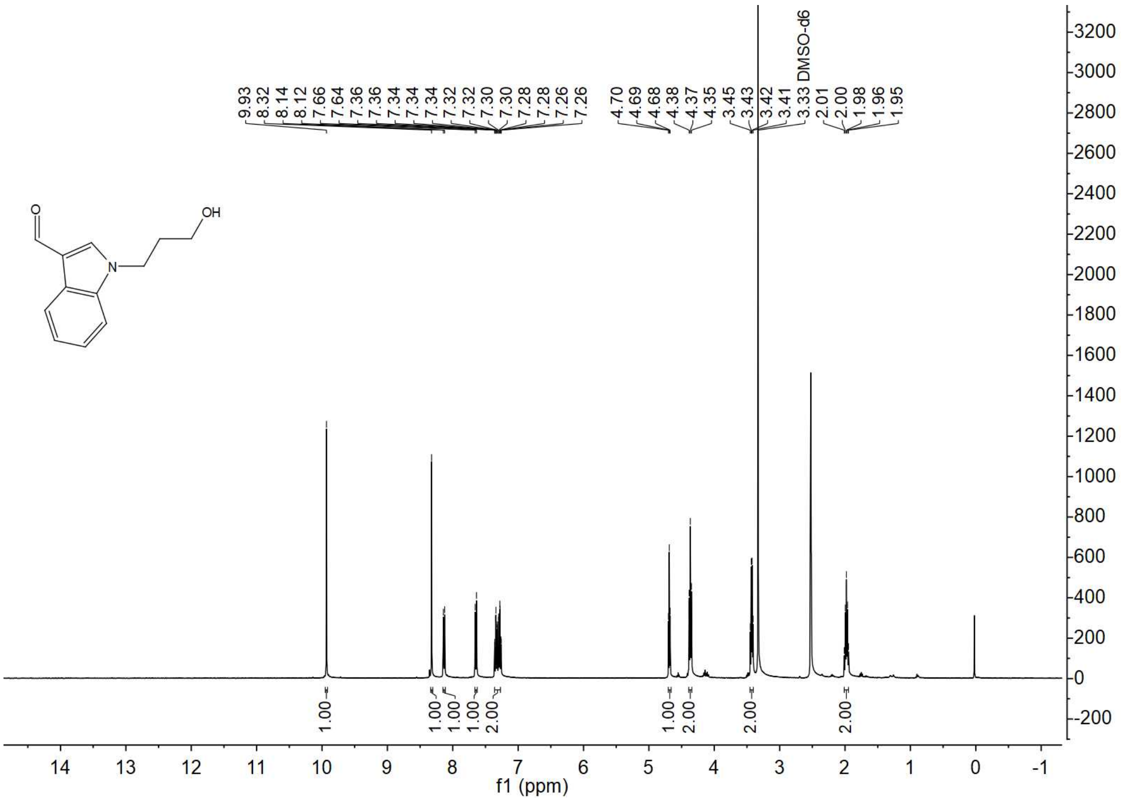

Figure S11. ^{1}H NMR spectrum of intermediate **6**.

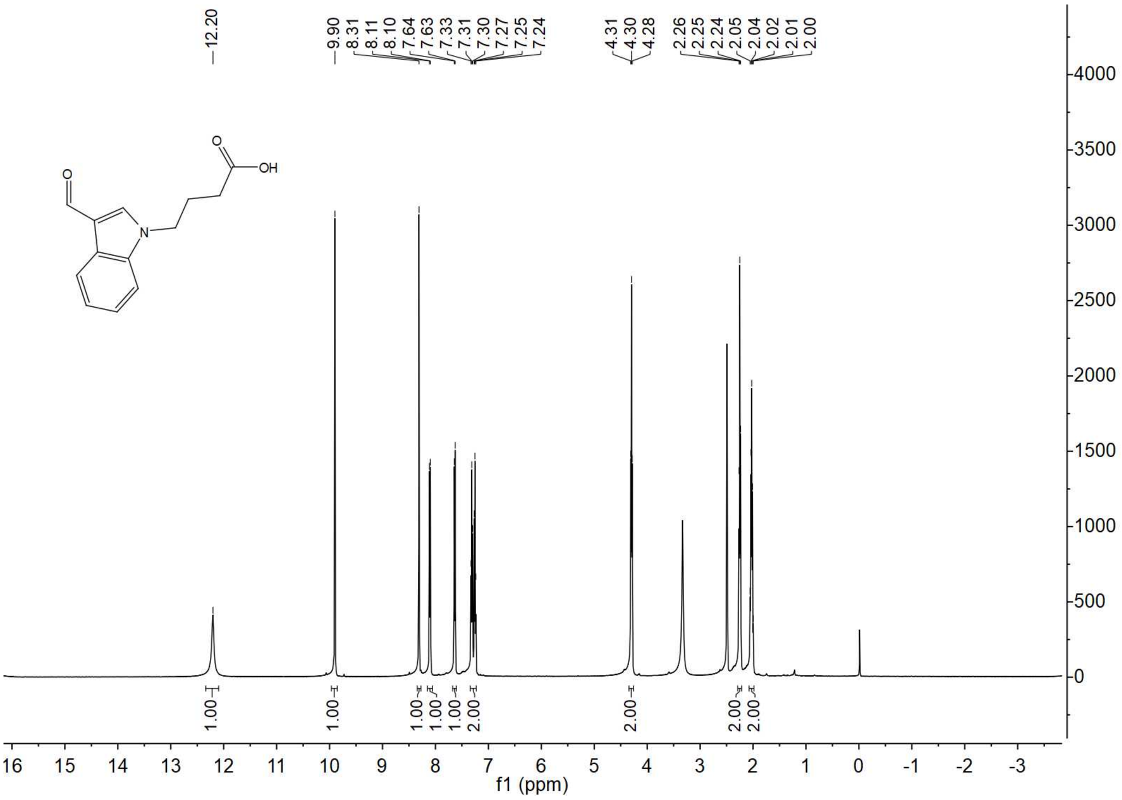

Figure S12. ^{1}H NMR spectrum of intermediate **7.**

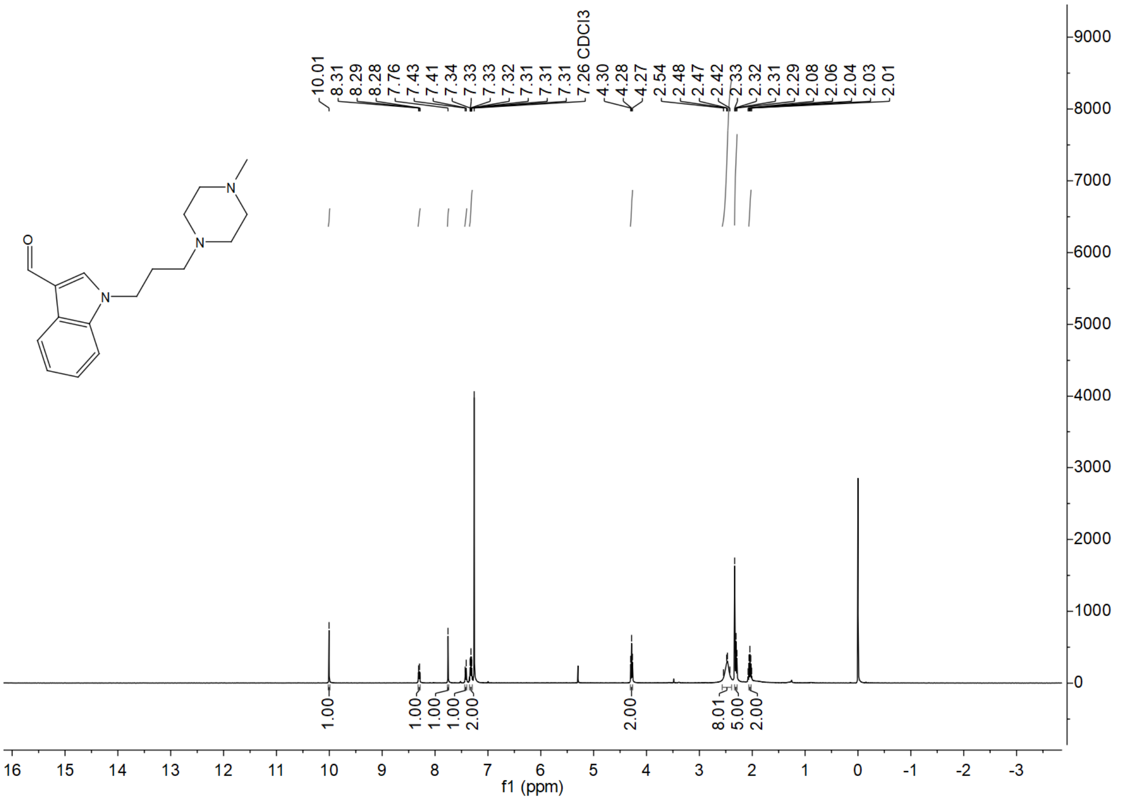

Figure S13. ^{1}H NMR spectrum of intermediate **8**.

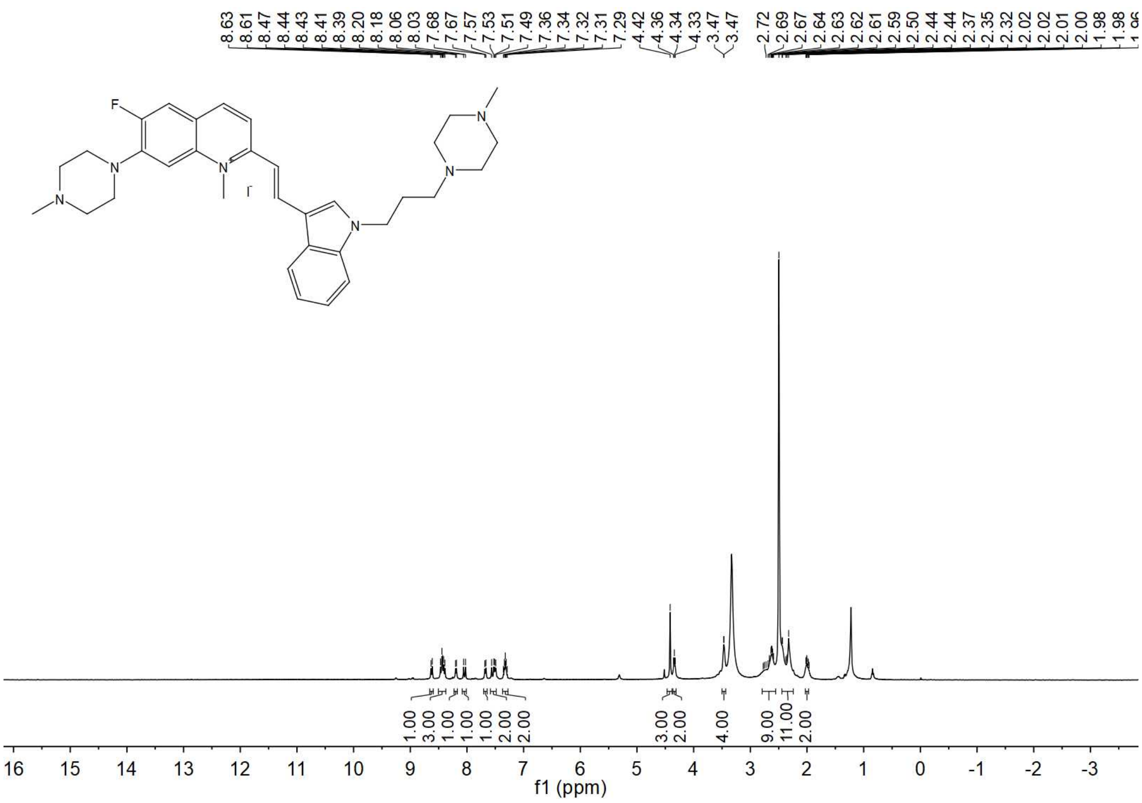

Figure S14. ^{1}H NMR spectrum of **QUID-2**.

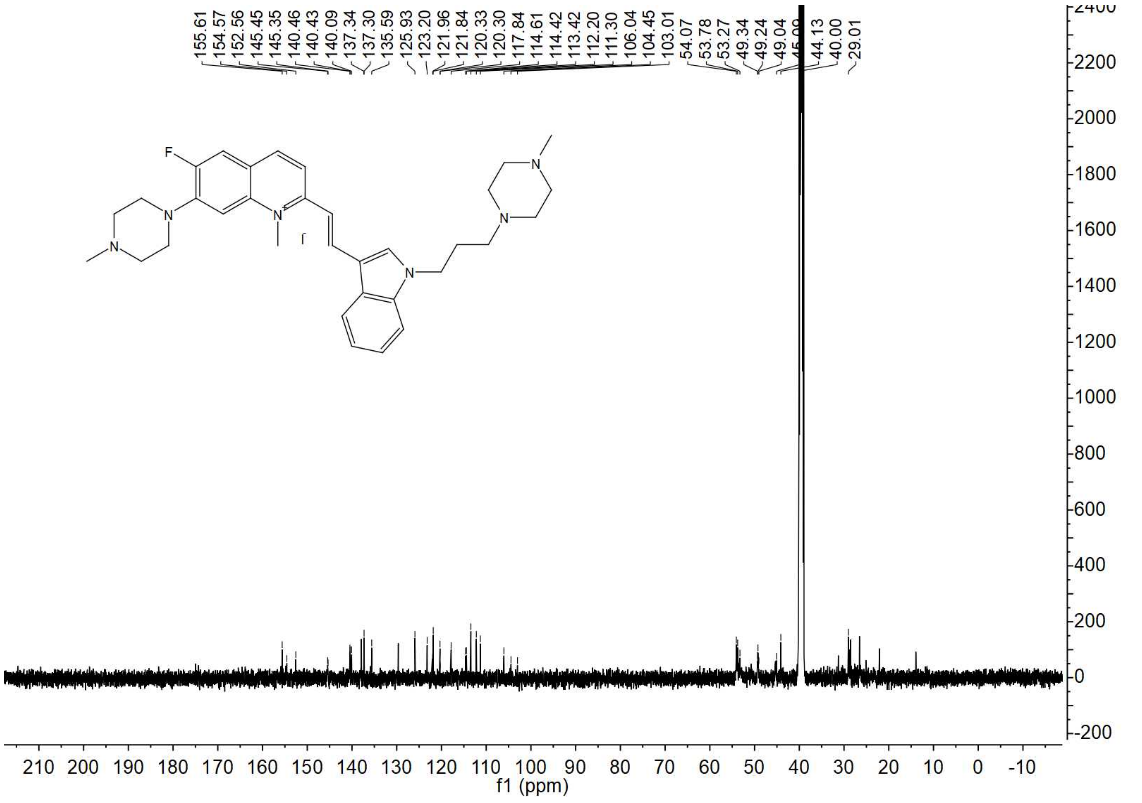

Figure S15. ^{13}C NMR spectrum of **QUID−2**.

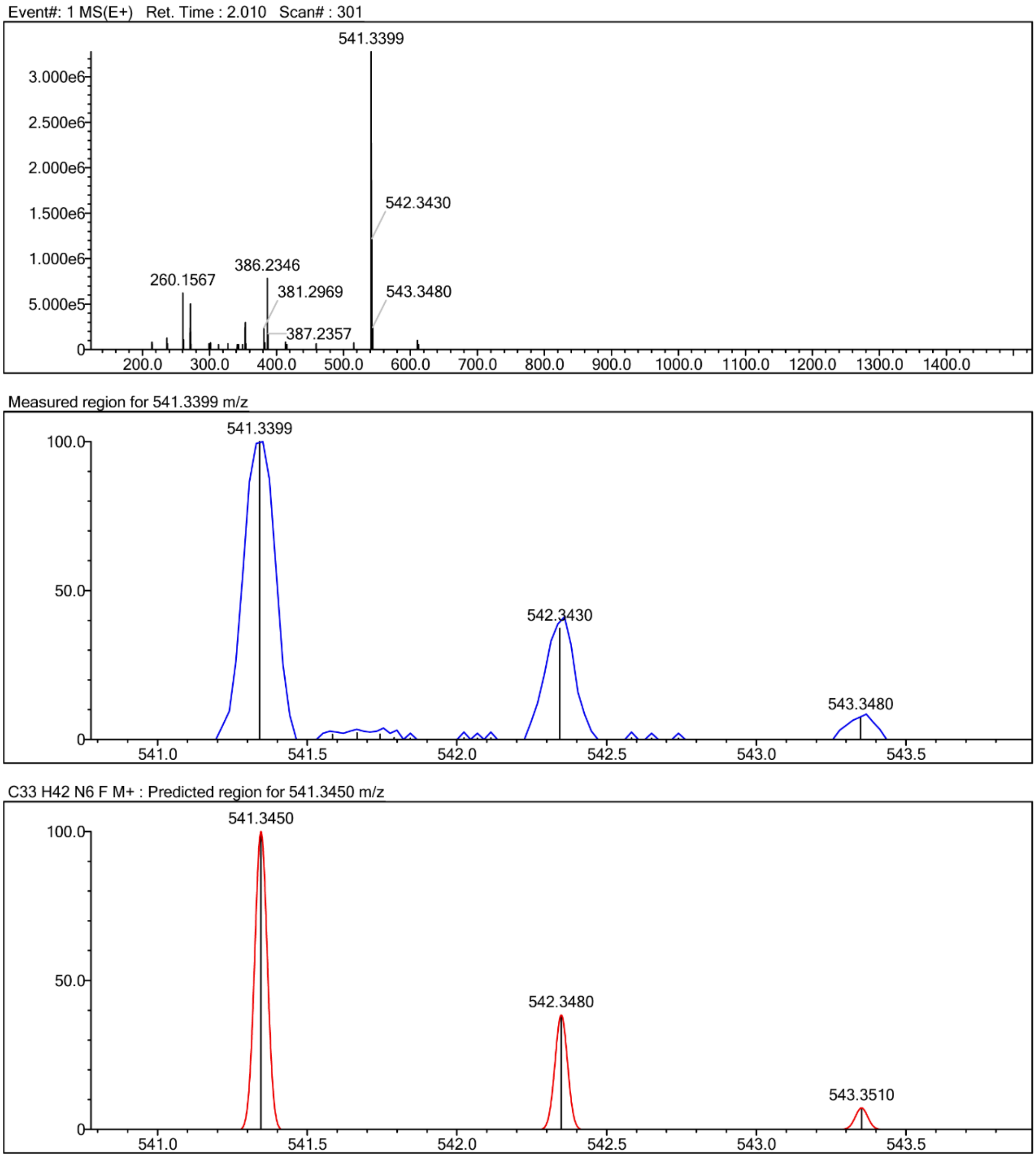

Rank	Score	Formula (M)	Ion	Meas. m/z	Pred. m/z	Df. (mDa)	Df. (ppm)	Iso	DBE
2	39.49	C33 H42 N6 F	M+	541.3399	541.3450	-5.1	-9.42	86.22	15.5

Figure S16. HRMS spectrum of **QUID−2**.

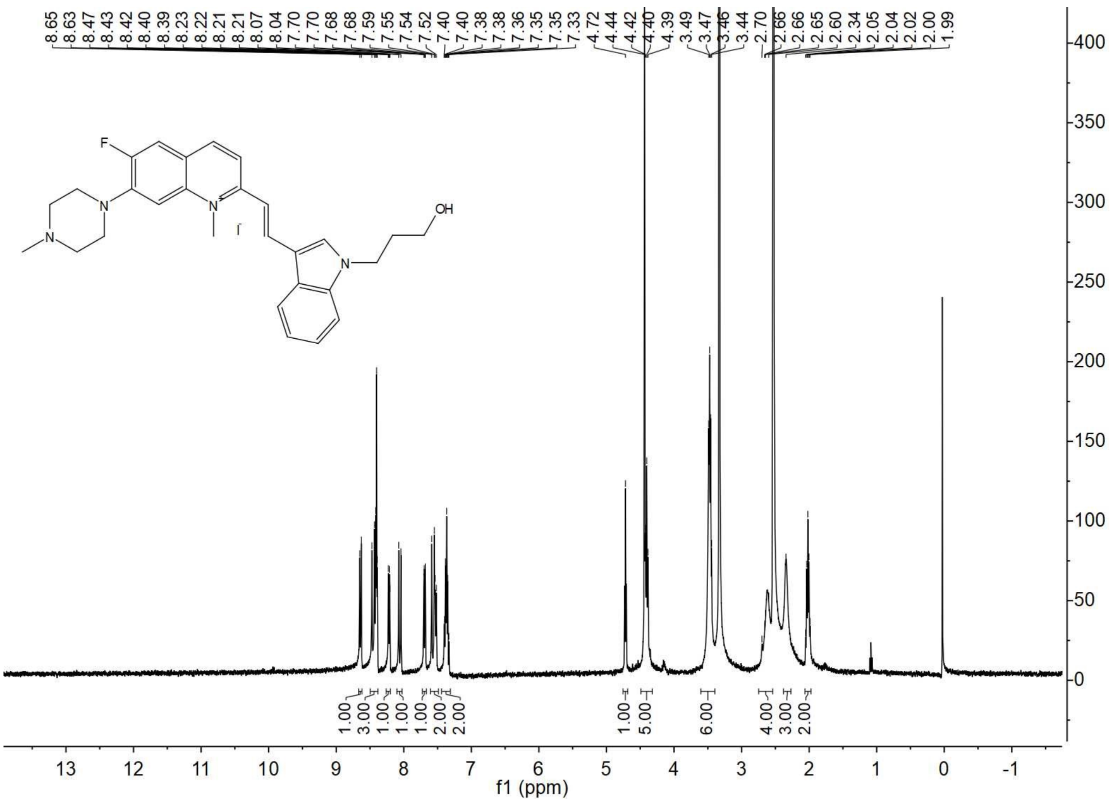

Figure S17. ^{1}H NMR spectrum of **QUID−3**.

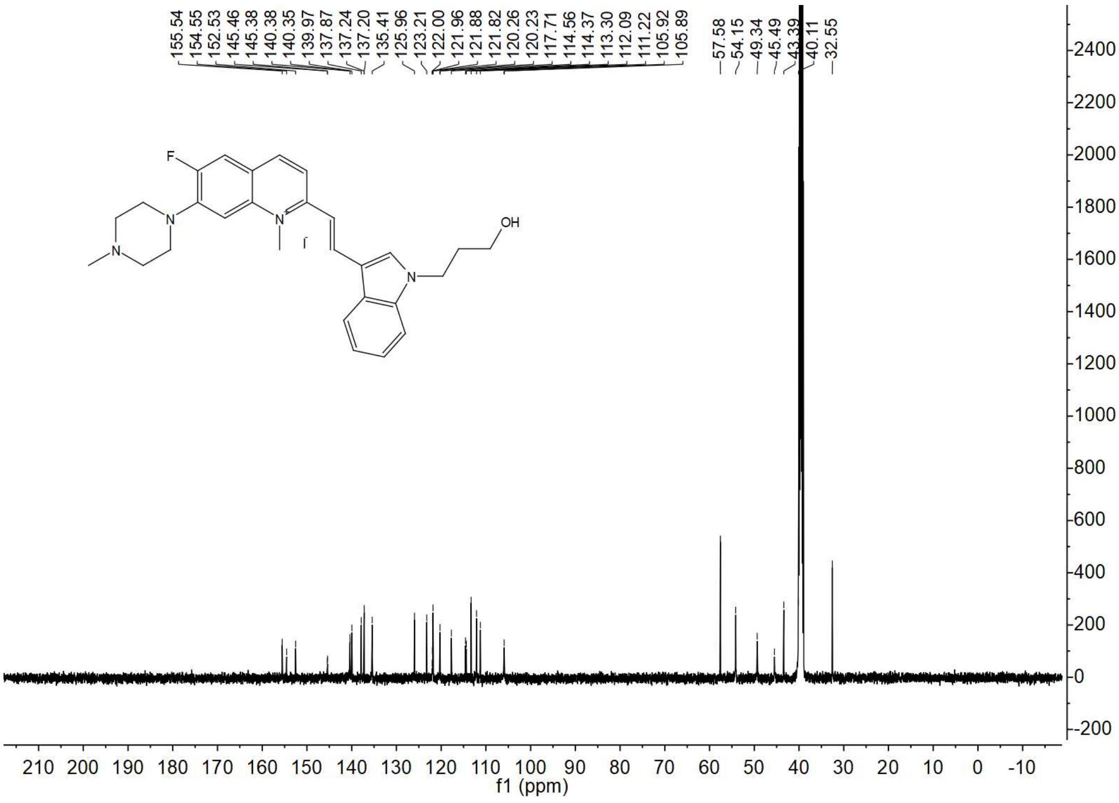

Figure S18. ^{13}C NMR spectrum of **QUID−3**.

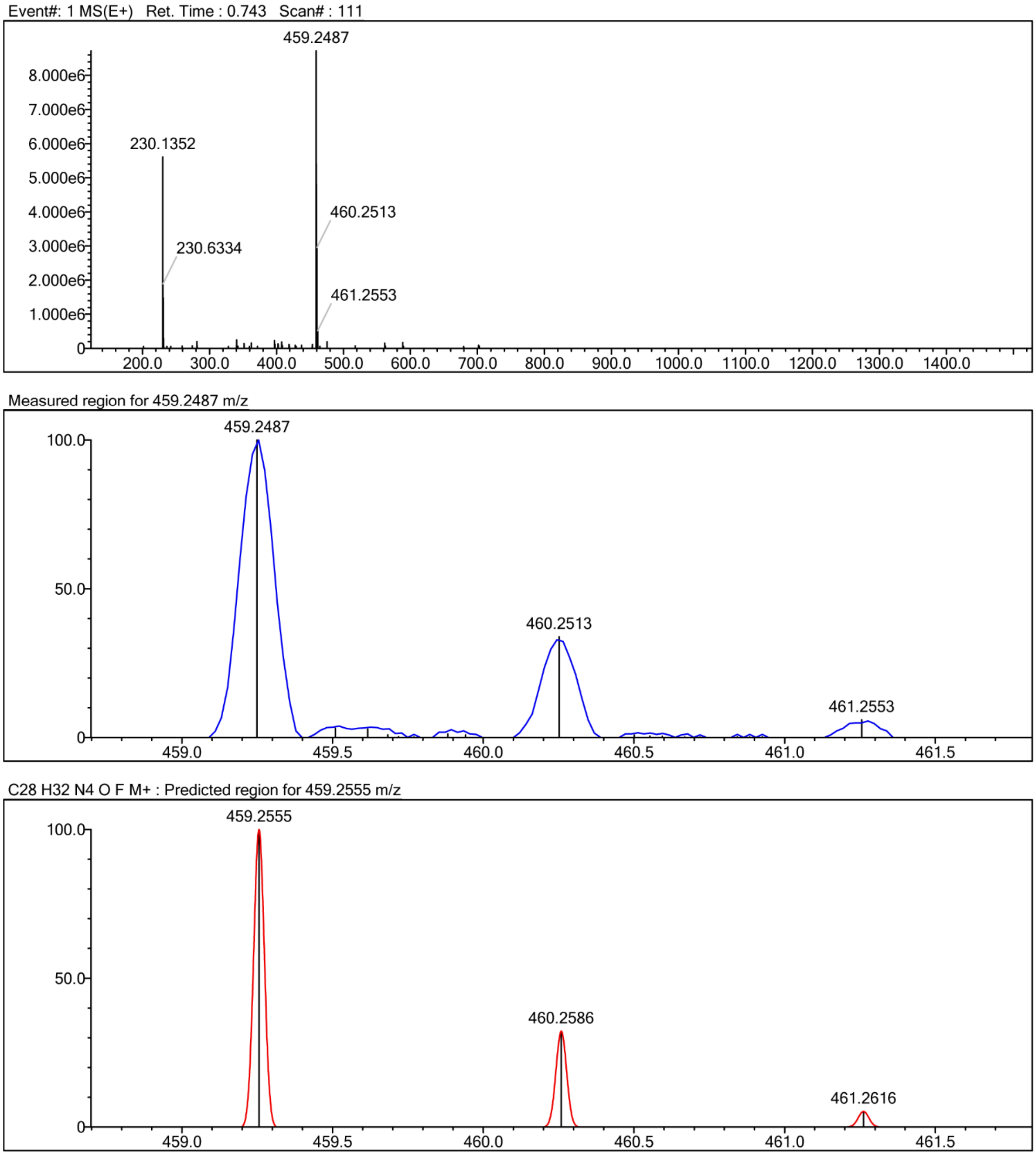

Rank	Score	Formula (M)	Ion	Meas. m/z	Pred. m/z	Df. (mDa)	Df. (ppm)	Iso	DBE
2	21.73	C28 H32 N4 O F	M+	459.2487	459.2555	-6.8	-14.81	79.97	14.5

Figure S19. HRMS spectrum of **QUID−3**.

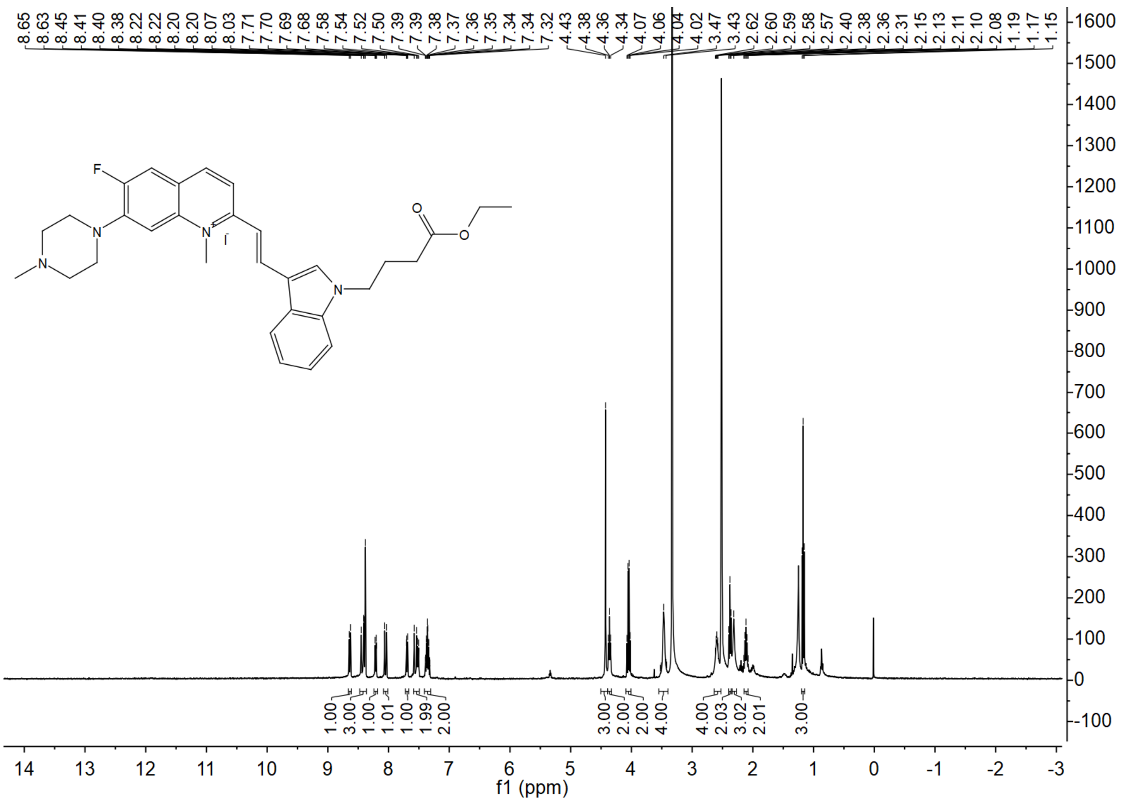

Figure S20. ^{1}H NMR spectrum of **QUID−4**.

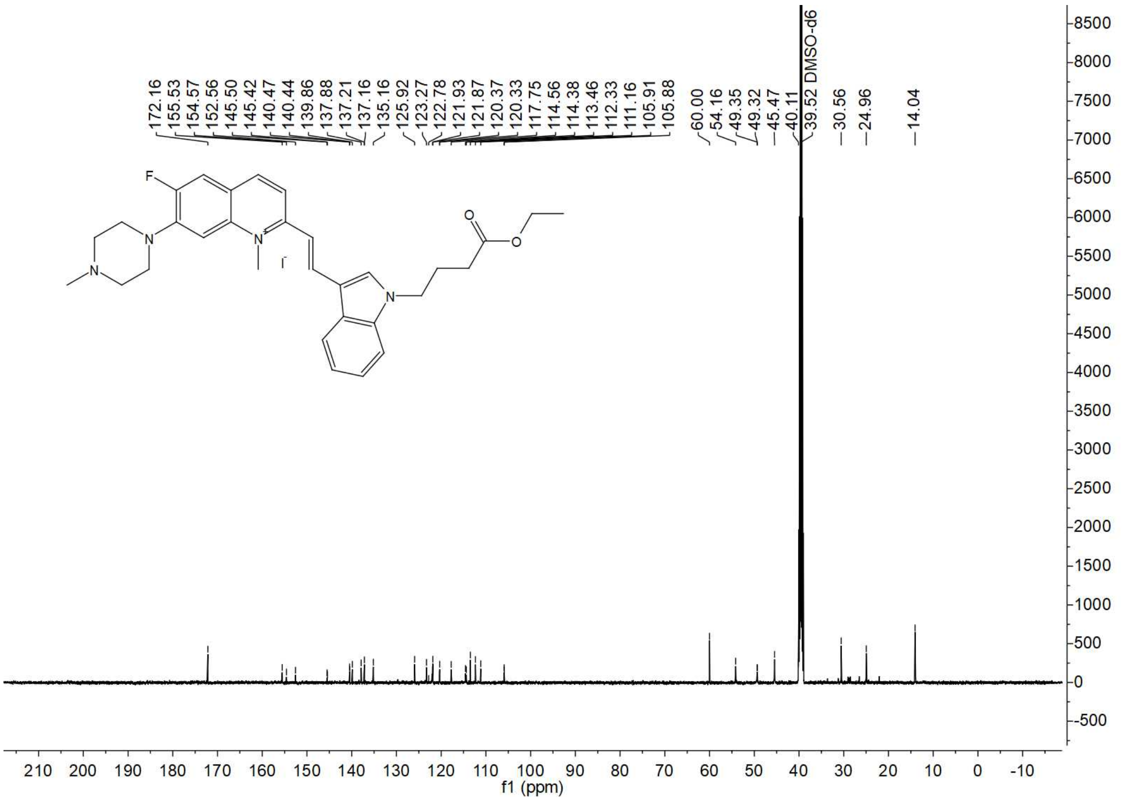

Figure S21. ^{13}C NMR spectrum of **QUID−4**.

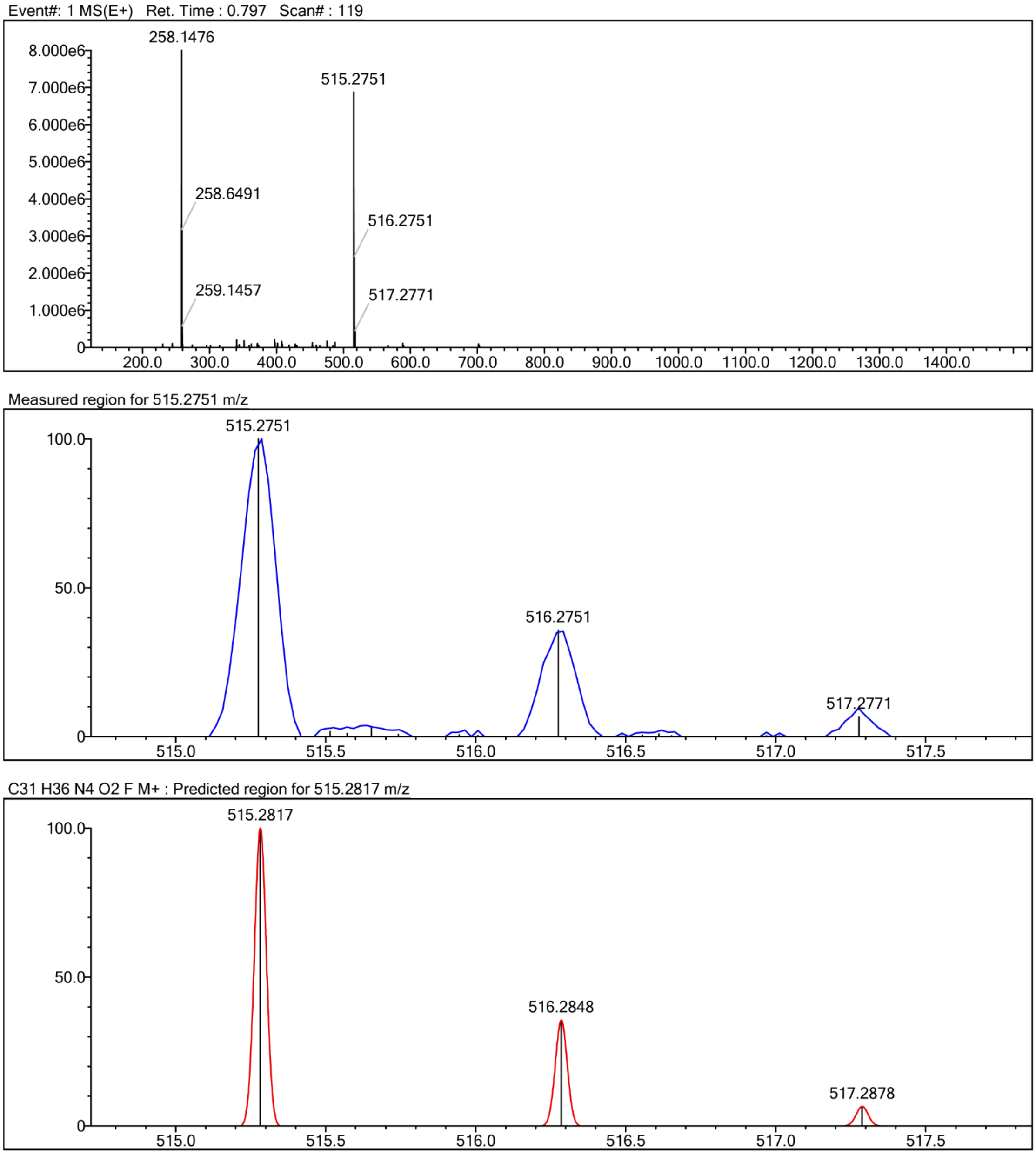

Rank	Score	Formula (M)	Ion	Meas. m/z	Pred. m/z	Df. (mDa)	Df. (ppm)	Iso	DBE
2	24.79	C31 H36 N4 O2 F	M+	515.2751	515.2817	-6.6	-12.81	76.26	15.5

Figure S22. HRMS spectrum of **QUID−4**.

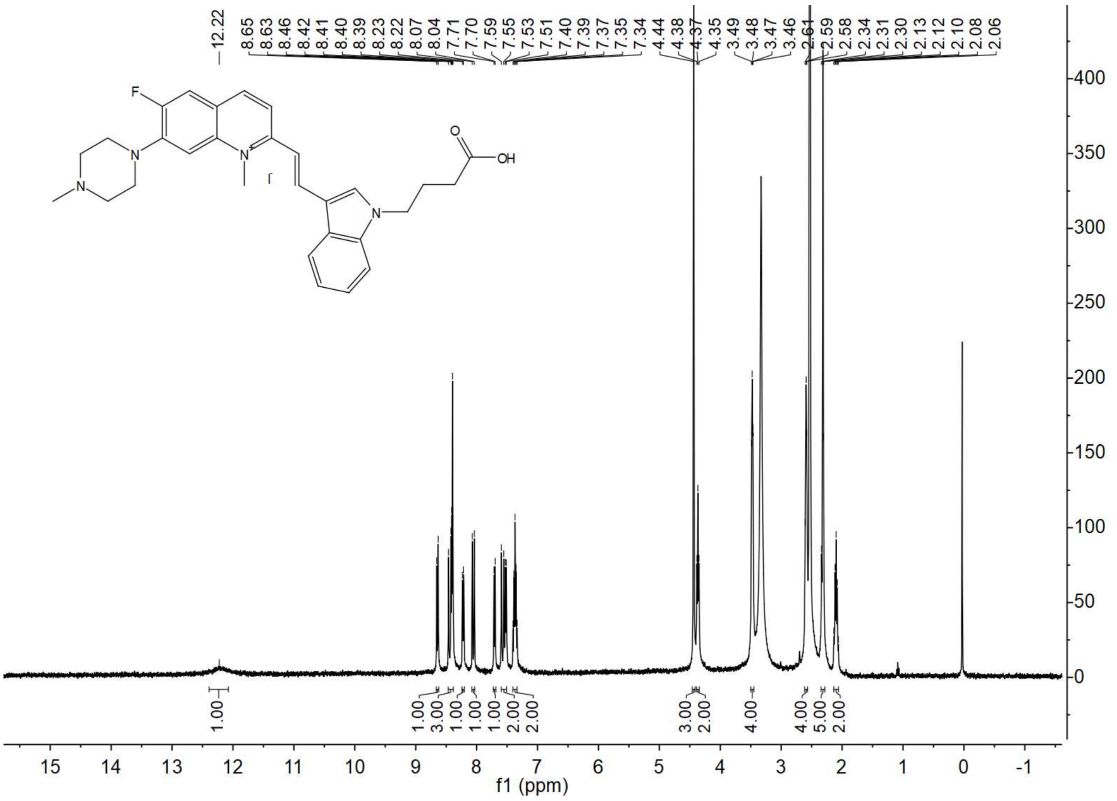

Figure S23. ^{1}H NMR spectrum of **QUID−5**.

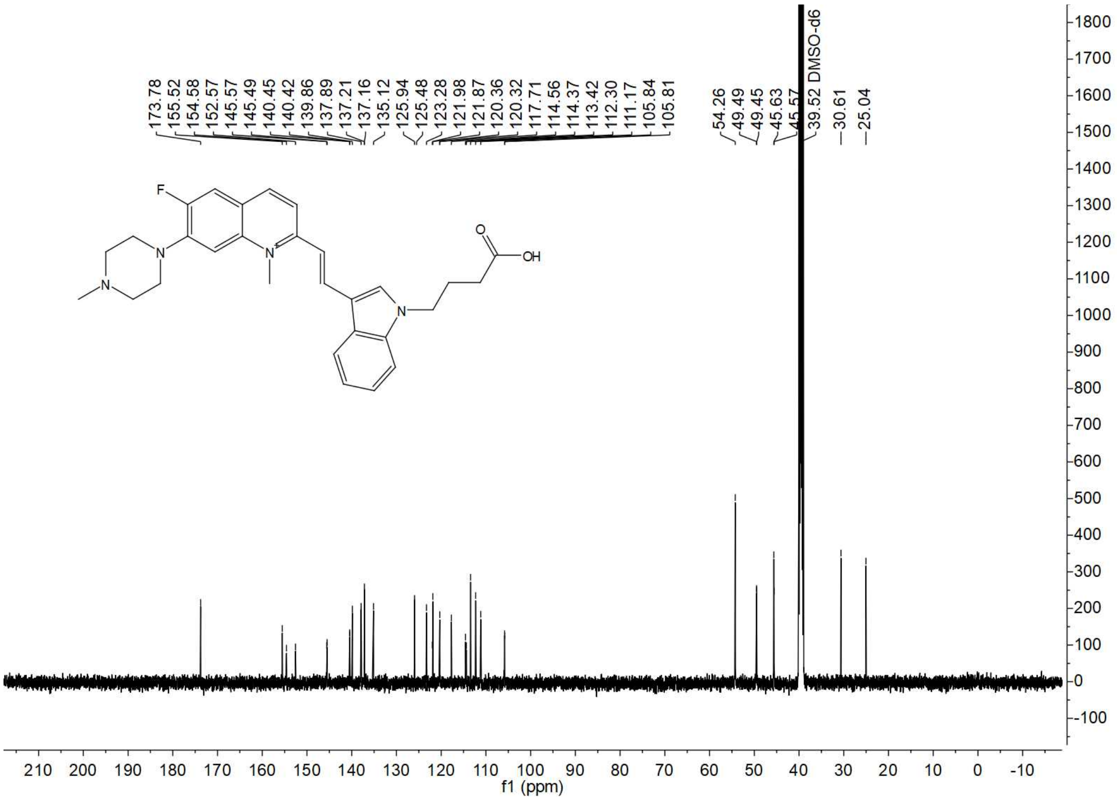

Figure S24. ^{13}C NMR spectrum of **QUID−5**.

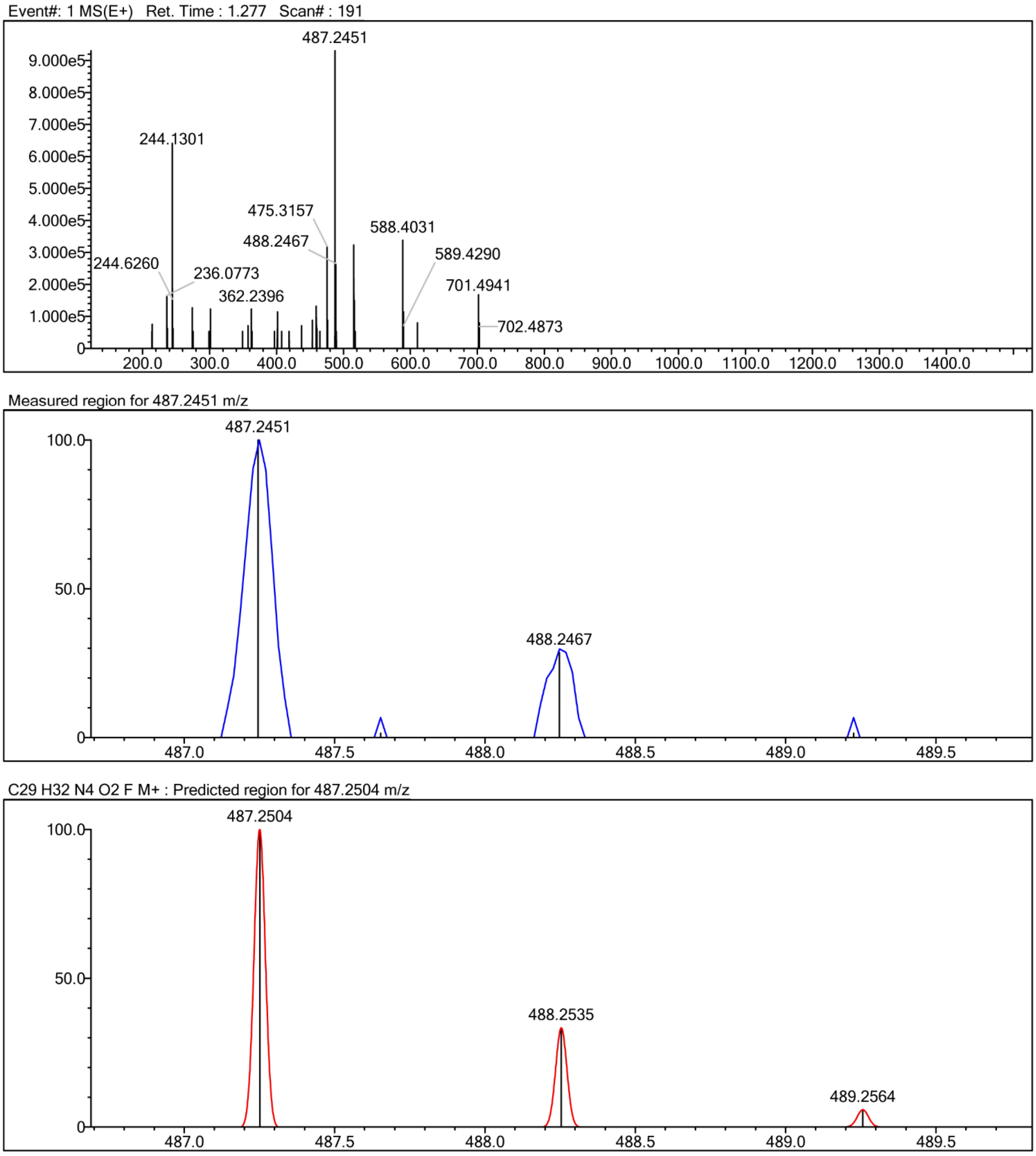

Rank	Score	Formula (M)	Ion	Meas. m/z	Pred. m/z	Df. (mDa)	Df. (ppm)	Iso	DBE
2	28.43	C29 H32 N4 O2 F	M+	487.2451	487.2504	-5.3	-10.88	75.51	15.5

Figure S25. HRMS spectrum of **QUID−5**.

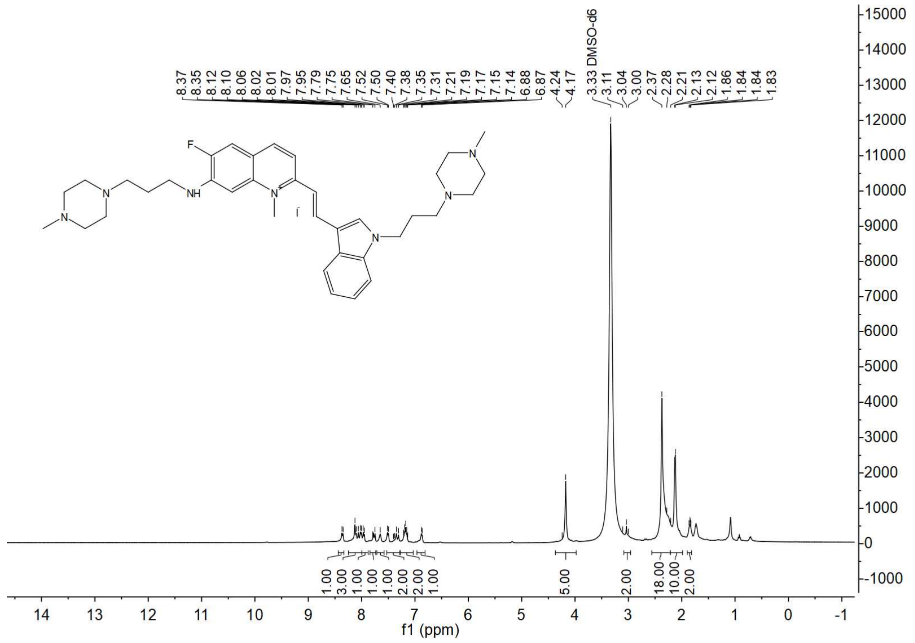

Figure S26. ^{1}H NMR spectrum of **QUID−6**.

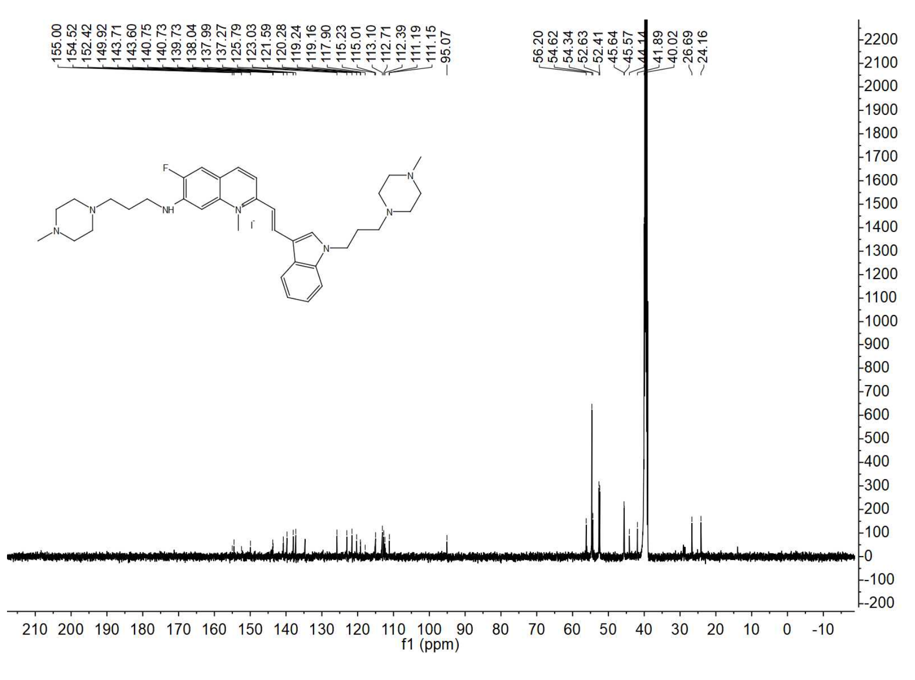

Figure S27. ^{13}C NMR spectrum of **QUID−6**.

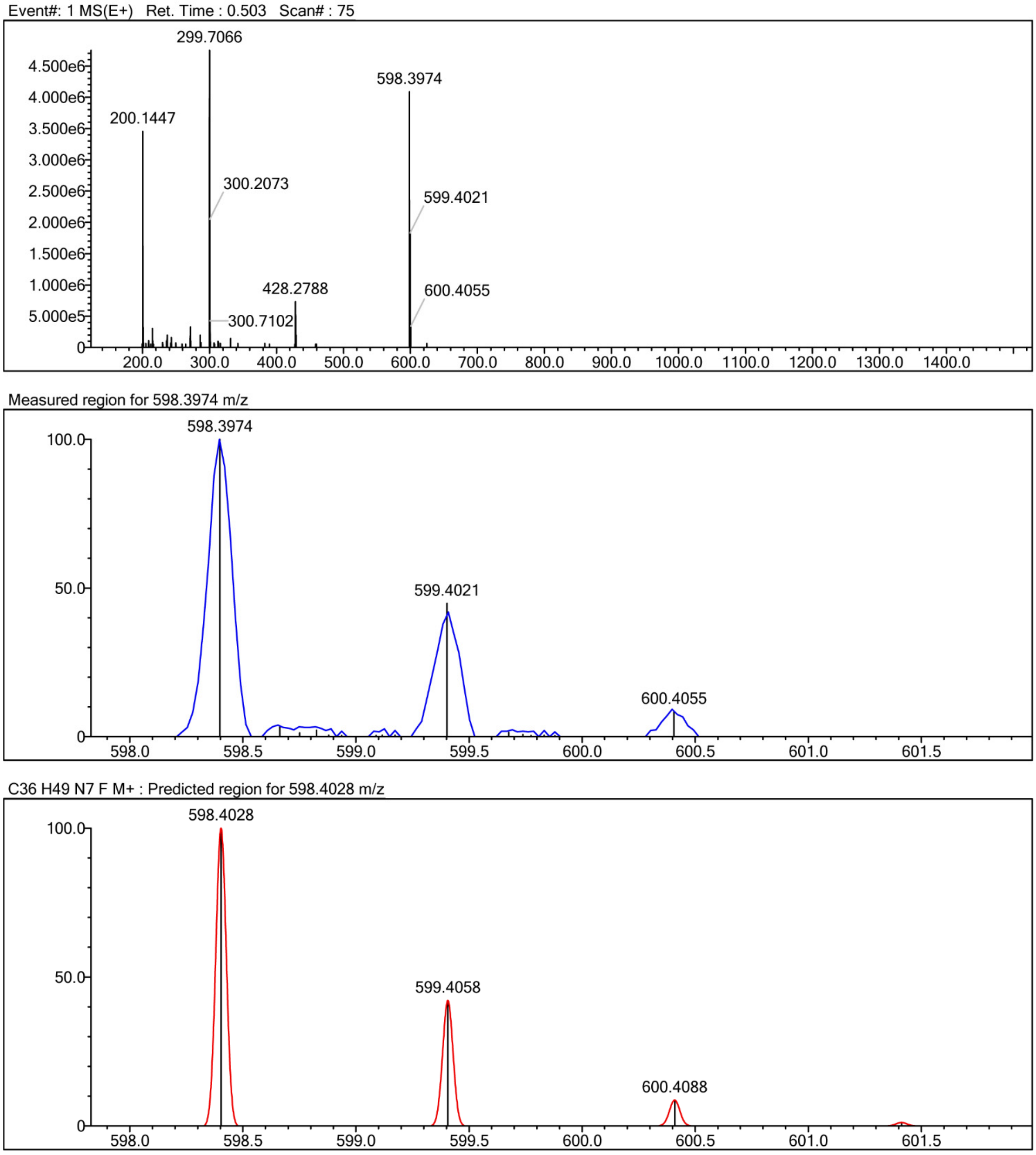

Rank	Score	Formula (M)	Ion	Meas. m/z	Pred. m/z	Df. (mDa)	Df. (ppm)	Iso	DBE
2	44.97	C36 H49 N7 F	M+	598.3974	598.4028	-5.4	-9.02	90.29	15.5

Figure S28. HRMS spectrum of **QUID−6**.
